# Stepwise Synthesis of *Tetra*-imidazolium Macrocycles and Their *N*-Heterocyclic Carbene Metal Complexes

**DOI:** 10.3389/fchem.2019.00270

**Published:** 2019-04-24

**Authors:** Zili Li, Nuchareenat Wiratpruk, Peter J. Barnard

**Affiliations:** Department of Chemistry and Physics, La Trobe Institute for Molecular Science, La Trobe University, Melbourne, VIC, Australia

**Keywords:** N-heterocycle carbene, macrocycle, anion receptor, N-heterocarbene-gold(I) complexes, tetra-imidazolium

## Abstract

A modular stepwise synthetic method has been developed for the preperation of *tetra*-imidazolium macrocycles. Initially a series of three *bis*(imidazolylmethyl)benzene precursors were alkylated with 1,2-dibromoethane to produce the corresponding *bis*-bromoethylimidazolium bromide salts. In the second step the *bis*-bromoethylimidazolium bromide salts were reacted with selected *bis*(imidazolylmethyl)benzene molecules to produce a series of two symmetrical and three asymmetrical *tetra*-imidazolium macrocycles. These *tetra*-imidazolium salts act receptors for anions and ^1^H-NMR titration studies were used to determine the association constants between two of the macrocycles and the halide anions chloride, bromide and iodide. The *tetra*-imidazolium salts are precursors for *N*-heterocyclic carbene (NHC) ligands and the corresponding silver(I), gold(I), and palladium(II) NHC complexes have been prepared. Varied structures were obtained, which depend on the chosen macrocyclic ligand and metal ion and in the case of the coinage metals Ag(I) and Au(I), mono, di, and hexanuclear complexes were formed.

## Introduction

**Figure 1:**
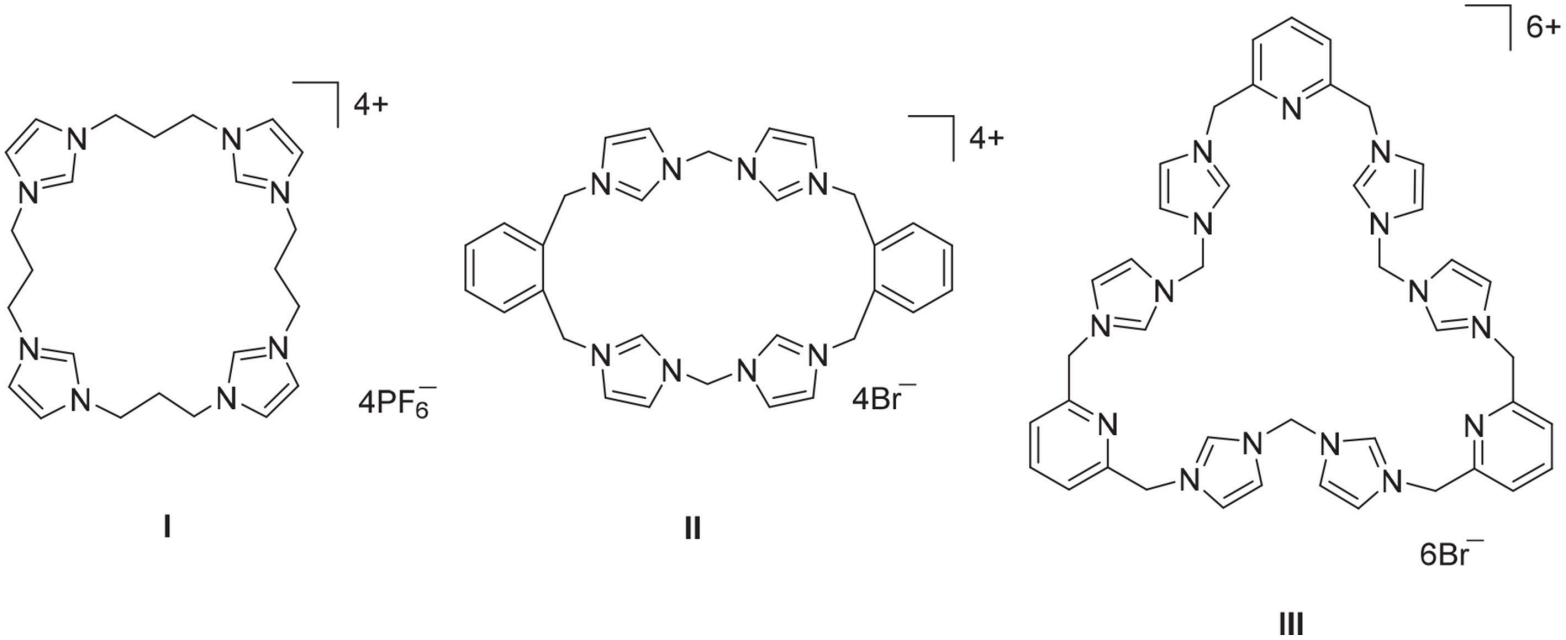
Polyimidazolium linked macrocyclic compound prepared by stepwise macrocyclization (I) and direct macrocyclization (II) and (III) methodologies.

Imidazolium linked macrocycles have attracted significant recent attention because of their capacity to act as anion receptors and to function as pro-ligands for the synthesis of *N*-heterocyclic carbene metal complexes. Due to the great importance of negatively charged anionic species in biology, the preparation of receptor molecules designed to recognize and sense anions is an area of great research interest (Beer and Gale, 2001; Gale, 2003; Martínez-Máñez and Sancenón, 2003). Imidazolium groups are now well-recognized for their favorable features for the generation of anion receptors, which result both from electrostatic and hydrogen bonding interactions (Alcalde et al., 1999, 2007; Chellappan et al., 2005; Wong et al., 2005; Yoon et al., 2006; Xu et al., 2010). For example, a series of *tetra*-imidazolium linked macrocyclic compounds e.g., **I** (Figure 1) were shown to bind to biologically relevant anions such as chloride and hydrogen sulfate (Wong et al., 2005; Serpell et al., 2011). Additionally, compounds of this type have been used as selective luminescent sensors for nucleic acids and nucleotide derivatives such as DNA, RNA, ATP, and GTP and to sense and image RNA in the living cells, as a result of strong C-H^+^···A^−^ hydrogen bonding interations (Neelakandan and Ramaiah, 2008; Ahmed et al., 2011, 2012; Shirinfar et al., 2013).

A range of imidazolium linked macrocyclic compounds have been previously reported and both direct macrocyclization (Bass et al., 2010; Altmann et al., 2015; Toure et al., 2016) and stepwise macrocyclization (Mesquida et al., 2013) processes have been previously described for the synthesis of compounds of this type. In the direct macrocyclization approach, equal quantities of a *bis*-imidazole precursor, and a dihaloalkane are combined under high dilution conditions to produce the desired macrocyclic compound. For example, the imidazolium linked macrocycles **II** and **III** were prepared using the direct macrocyclization approach (Hahn et al., 2008; Schulte to Brinke et al., 2013). This approach can be limited due to unintended reactivity between the precursor compounds, which can produce complex reaction mixtures and poor yields of the desired product. Alternatively, stepwise macrocyclization can be used for the synthesis of polyimidazolium linked macrocyclic compounds (Schulte to Brinke and Hahn, 2015) and the synthesis of the *tetra*-imidazolium macrocycle **I** was achieved via sequential alkylation of *bis*-imidazolium precursors (Wong et al., 2005).

**Figure 2:**
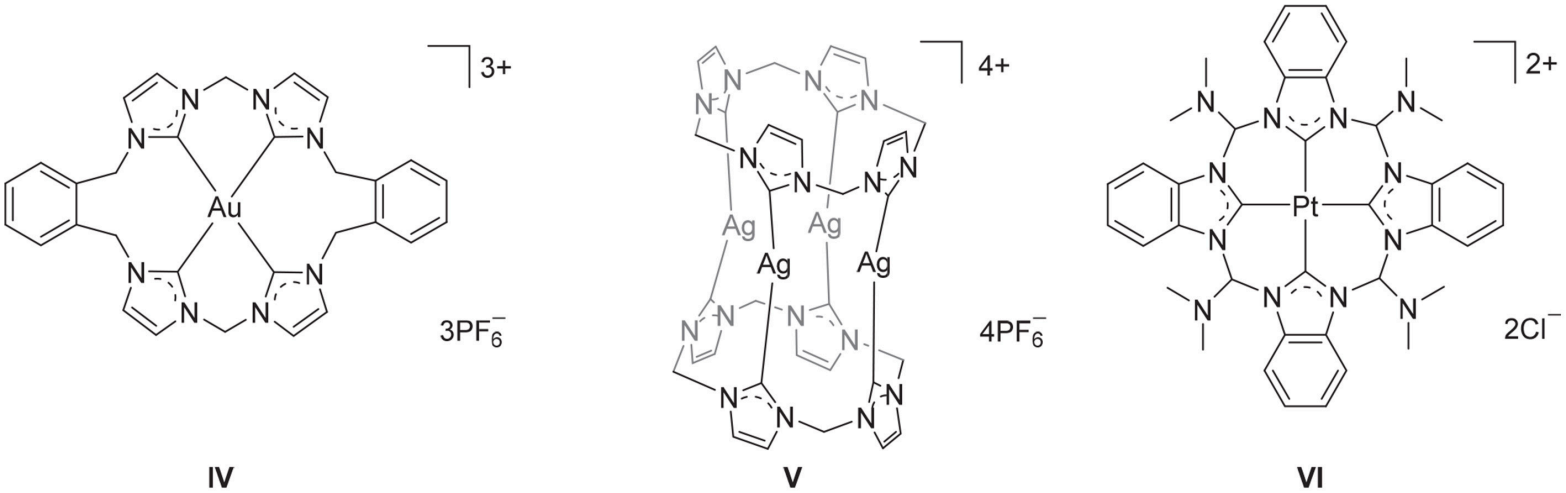
Selected metal complexes derived from macrocyclic imidazolium pro-ligands.

Polyimidazolium linked macrocycles have also been utilized as precursors for *N*-heterocyclic carbene metal complexes and previously the Au(III) complex **IV** (Figure 2) was prepared the reaction of KAuCl_4_ with a macrocyclic *tetra*-imidazolium salt **II** (Mageed et al., 2017). Additionally, dinuclear Au(II) and mixed valence Au(I)/Au(III) complexes of have been prepared by the oxidation of dinuclear Au(I) complexes of macrocyclic NHC ligands (Mageed et al., 2018). In the past decade, a number of Ag(I) complexes of macrocyclic NHC ligands that display wide range of structures have been reported (Mckie et al., 2007; Hahn et al., 2008; Schulte to Brinke et al., 2013; Altmann et al., 2016; Fei et al., 2017; Lu et al., 2017, 2018). For example, a sandwich like tetranuclear Ag(I) complex featuring two *tetra*-NHC ligands was prepared **V** (Figure 2) and this complex was utilized as a precursor for the preparation of Au(I), Ni(II), Pd(II), and Pt(II) complexes (Altmann et al., 2016). In addition, macrocyclic *tetra*-NHC ligands have been shown to act as tetradentate ligand for square-planer metals such as Pd(II), Ni(II), Cu(I), and Pt(II) (Fei et al., 2017) and the interesting square-planar Pt(II) complex **VI** (Figure 2) was prepared by metal-template reaction from the *tetrakis*(trimethylphosphane)platinum(II) triflate and 2-azidophenylisocyanide (Hahn et al., 2005). The biological properties of metal complexes of NHC-based cyclophane and macrocyclic ligands have also been of significant recent interest and Ag(I) and Au(I) complexes have been shown to possess potent antimicrobial and anticancer activities, respectively (Aweda et al., 2013; Shah et al., 2013; Nomiya et al., 2018; Pöthig et al., 2018). Youngs et al. have been particularly active in this field (Hindi et al., 2009; Johnson et al., 2017) and have described the potent antimicrobrial properties of a Ag(I)-NHC complexes of cyclophane ligands (Melaiye et al., 2005). Additionally, a series of Au(I) complexes of related cyclophane-based NHC ligand systems were shown to be selectively toxic to cancer cells as a result of an antimitochondrial mechanism (Barnard et al., 2004, 2006). Meyer and more recently Kühn have also extended the application of macrocyclic tetra-NHC ligands to the synthesis of iron complexes, that provide fascinating models of reactive intermediates that are generated in the catalytic cycles of a range of heme and non-heme iron enzymes (Meyer et al., 2013; Anneser et al., 2015).

As imidazolium linked macrocycles offer the potential for the generations of novel sensors for biologically significant anions and as precursors for NHC metal complexes of metals that are well-known for their biological properties, we became interested in developing new strategies for the synthesis of compounds of this type. In the present paper, we report a novel modular stepwise synthetic approach for the synthesis of *tetra*-imidazolium macrocycles. This approach involves the initial synthesis of *bis*-bromoethylimidazolium bromide precursors, which can then be utilized for the formation of either symmetrical or asymmetrical *tetra*-imidazolium macrocycles. These *tetra*-imidazolium salts bind anions in solution and association constants between two of the macrocycles and the halide anions chloride, bromide, and iodide were determined for two macrocycles. A range of silver(I), gold(I), and palladium(II) NHC complexes have been prepared from these pro-ligands.

## Results and Discussion

### Synthesis of *Tetra*-imidazolium Macrocycles

**Scheme 1:**
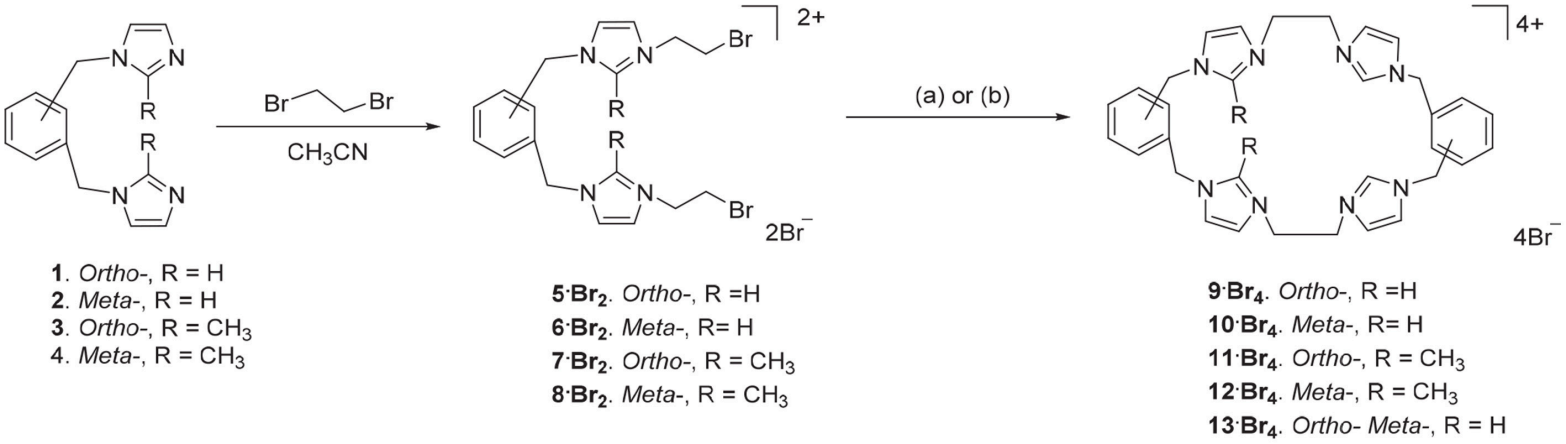
Synthesis of *tetra*-imidazolium macrocyclic compounds **9·Br**_**4**_**-13·Br**_**4**_. (a) DMF/CH_3_CN 1:4, 110°C. (b) Bu_4_N·Br (6.0 eq.), DMF/CH_3_CN 1:4, 110°C.

The *ortho*-phenylene linked *tetra*-imidazolium macrocycle **9·Br**_**4**_ was prepared via a stepwise macrocyclization procedure. Initially, 1,2-*bis*(imidazolylmethyl)benzene **1**(Baker et al., 2001) was alkylated with an excess of 1,2-dibromoethane (30 equivalents) to obtain the *bis*-bromoethylimidazolium bromide salt **5·Br**_**2**_ in a moderate yield (Scheme 1). The ^1^H-NMR spectrum of **5·Br**_**2**_ shows the imidazolium C2-H proton resonates as a singlet signal at 9.50 ppm, while the ethylene group protons resonate as two upfield shifted triplet signals at 4.00 and 4.68 ppm. The macrocycle **9·Br**_**4**_, was prepared by heating an equimolar mixture of **5·Br**_**2**_ and **1** in a solvent system consisting of a 1:4 mixture of DMF and acetonitrile under high dilution conditions (Scheme 1) and was isolated in a moderate yield of 29.7%. Compound **9·Br**_**4**_ gave a simple ^1^H-NMR spectrum consistent with its high symmetry (point group *D*_2h_) with downfield shifted signals for the imidazolium protons which resonated at 9.45 (C2-H) and 7.98 and 7.88 ppm (C4-H/C5-H). The protons of the ethylene linker groups resonated as a singlet signal at 4.81 ppm. A similar method was adopted for the synthesis of the *meta*-phenylene linked macrocycle **10·Br**_**4**_ from a mixture of **2** and **6·Br**_**2**_ (Scheme 1).

With the successful synthesis of the symmetrical macrocycles **9·Br**_**4**_ and **10·Br**_**4**_, we were interested in further exploring the versatility of our stepwise synthetic methodology with the synthesis of asymmetric *tetra*-imidazolium salts. In initial studies, reaction of either **3** with **5·Br**_**2**_ or **1** with **7·Br**_**2**_ (Scheme 1) under high dilution condition did not successfully produce the desired asymmetrical *tetra*-imidazolium salts. Additionally, attempts to use *tetra*-n-butylammonium bromide (Bu_4_N·Br) as a templation reagent in the reaction of **3** with **5·Br**_**4**_ also did not give the desired product. By contrast, the reaction of compound **1** with **7·Br**_**2**_ under high dilution condition in presence of Bu_4_N·Br gave the desired product **11·Br**_**4**_ in a low yield. The ^1^H-NMR spectrum of **11·Br**_**4**_ showed the imidazolium C2-H proton as singlet signal at 9.76 ppm and the methyl group on the imidazolium C2 carbon resonated as a singlet signal at 2.64 ppm. In addition, consistent with the lower symmetry structure (point group *C*_2h_) the benzylic proton resonates as two singlet signals at 5.47 and 5.52 ppm. The asymmetrical pro-ligands **12·Br**_**4**_ and **13·Br**_**4**_ were prepared in a similar manner from the reaction of either **2** and **8·Br**_**2**_ or **2** and **5·Br**_**2**_, respectively, in presence of Bu_4_N·Br. Again, the ^1^H-NMR spectrum for **13·Br**_**4**_ is consistent with the lower symmetry structure, with the C2-H protons resonating as two singlet signals at 8.99 and 9.13 ppm.

### Synthesis of Ag(I), Au(I), and Pd(II) Complexes

**Scheme 2:**
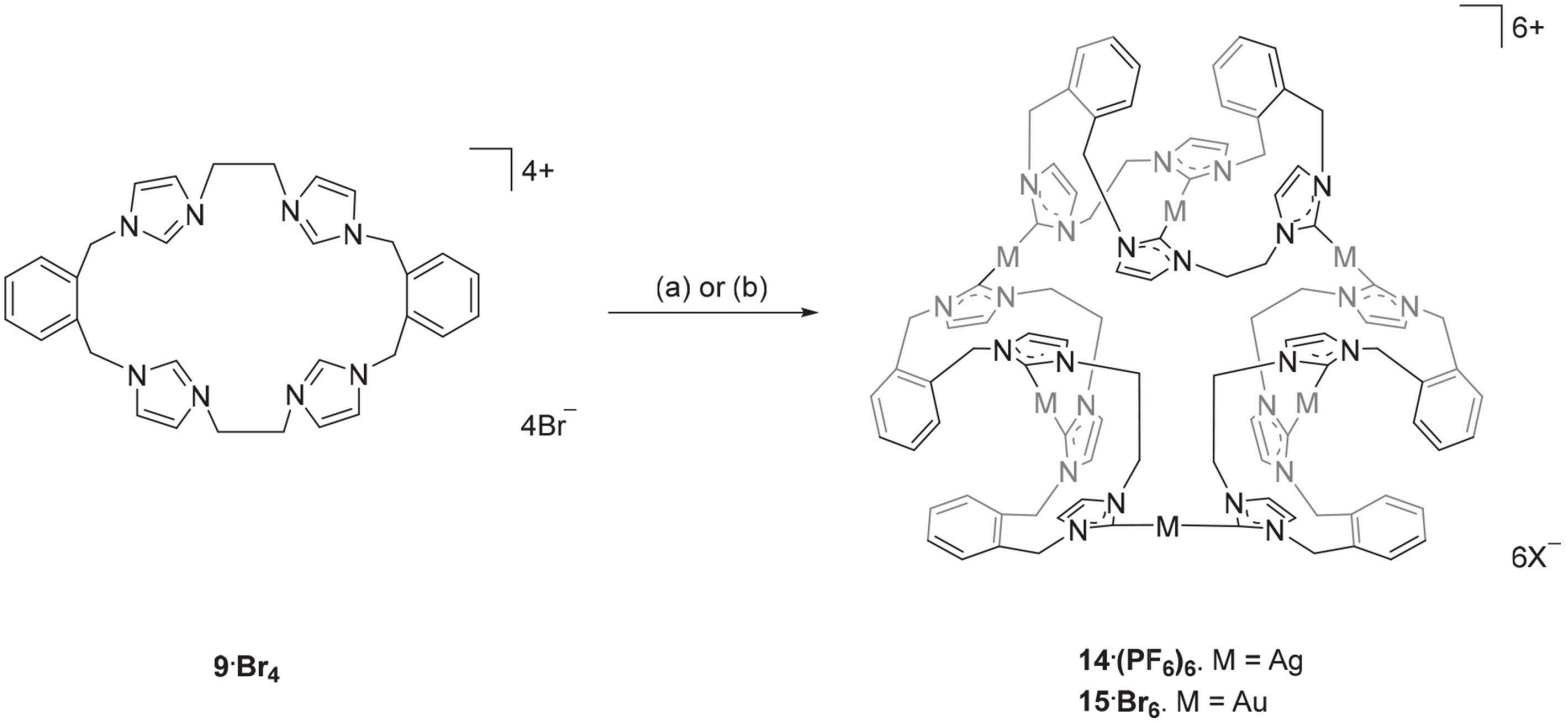
Synthesis of the Ag(I) and Au(I) complexes derived from pro-ligand **9·Br**_**4**_. (a) Ag_2_O (4.0 eq.), DMF, 50°C, 3 d. (b) (THT)AuCl (2.2 eq.), NaOAc, DMF, 110°C, 1 h.

The Ag(I) complex **14·(PF**_**6**_**)**_**6**_ was prepared by the reaction of pro-ligand **9·Br**_**4**_ with Ag_2_O in DMF with the exclusion of light (Scheme 2) and product was isolated as a white crystalline solid in a yield of 21%. The ^1^H-NMR spectrum of **14·(PF**_**6**_**)**_**6**_ showed no imidazolium C2-H proton signal, indicating that the C2 carbon is deprotonated and coordinated to the metal center as a carbene. The ortho-substituted phenyl-linker group protons resonate as two set of doublets (5.68 and 6.94 ppm) and triplet (7.17 and 7.52 ppm) signals, consistent with a more complex magnetic environment for the Ag(I) complex when compared to the pro-ligand. The ^13^C-NMR spectrum for **14·(PF**_**6**_**)**_**6**_ revealed a downfield shifted signal at 182 ppm, for which ^107^Ag-^13^C (d, ^1^*J* = 182.32 Hz) and ^109^Ag-^13^C (d, ^1^*J* = 209.99 Hz) couplings were observed, which is also consistent with coordination of the C2 carbon to Ag(I). The high-resolution mass spectrum for **14·(PF**_**6**_**)**_**6**_ produced a series of peaks consistent with a hexanuclear structure with the general formula [Ag_6_L_3_]^6+^ (where L is the macrocyclic *tetra*-carbene ligand). For example, a peak was observed at m/z = 372.0334, which corresponds to the formula [C_96_H_96_N_24_Ag_6_]^6+^ (calculated = 372.0419).

**Scheme 3:**
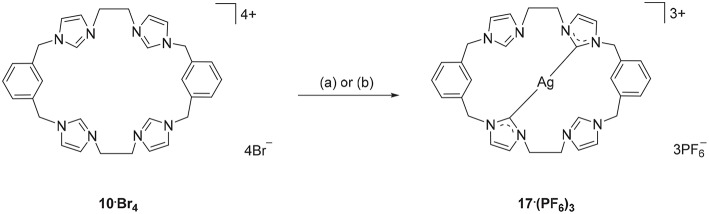
Synthesis of Ag(I) complex **17·(PF**_**6**_**)**_**3**_ from the pro-ligand **10·Br**_**4**_. (a) Ag_2_O (4.0 eq.), DMF, 70°C, 3 d. (b) AgNO_3_ (4.0 eq.), NH_4_OH (eq.), CH_3_CN, RT, overnight.

Using a similar approach, the Ag(I) complex **17·(PF**_**6**_**)**_**3**_ was prepared from the pro-ligand **10·Br**_**4**_, however for this compound the ^1^H-NMR spectrum showed a signal at 8.37 ppm, consistent with the C2-H proton being present in the complex. However, the ^13^C-NMR spectrum of **17·(PF**_**6**_**)**_**3**_ showed a downfield shifted signal at 180.4 ppm that displays ^107^Ag-^13^C (d, ^1^*J* = 183.58 Hz) and ^109^Ag-^13^C (d, ^1^*J* = 211.24 Hz) couplings, indicating coordination of the imidazole C2 carbon to Ag(I). These NMR results are consistent with a mononuclear complex, where two of the imidazole units are coordinated to the metal, while the other two remain uncoordinated imidazolium units (Scheme 3). To further investigate this result, a different synthetic method was undertaken where the pro-ligand **10·(PF**_**6**_**)**_**4**_ was reacted with AgNO_3_ in present of NH_4_OH, however the same mononuclear Ag(I) complex was obtained (Scheme 3).

**Scheme 4:**
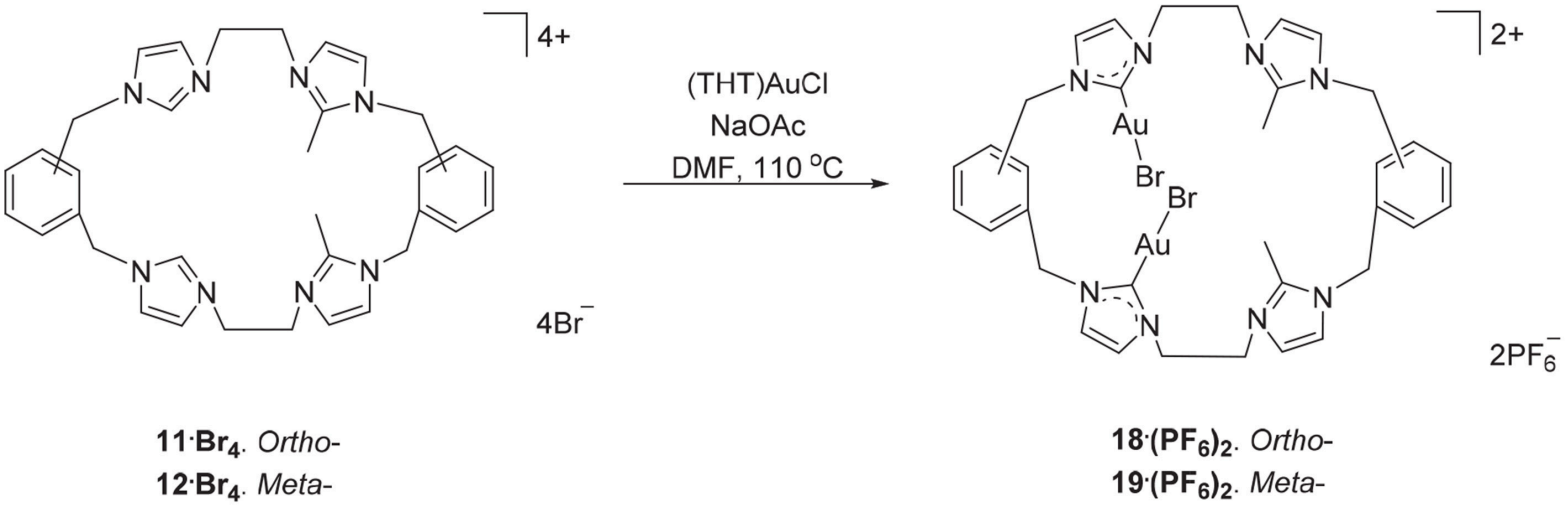
Synthesis of dinuclear Au(I) complex derived from **11·Br**_**4**_ to **12·Br**_**4**_.

The hexanuclear Au(I) complex **15·Br**_**6**_ was prepared by the reaction of **9·Br**_**4**_ with (THT)AuCl in present of the mild base sodium acetate (Scheme 2) and the complex was obtained as an off-white solid in 54.2% yield. The same approach was used in an attempt to prepare the Au(I) complex of pro-ligand **10·Br**_**4**_, however no complex could be isolated from the reaction mixture. In the next set of reactions, the pro-ligand **11·Br**_**4**_ (with both normal and C2-blocked imidazolium groups) was reacted with (THT)AuCl in presence of sodium acetate. It was anticipated that this ligand might produce a complex displaying both “normal” and “abnormal” NHC coordination modes, however the dinuclear Au(I) complex **18·(PF**_**6**_**)**_**2**_ was obtained, which displayed only the “normal” NHC coordination mode (Scheme 4). Using the same method, a dinuclear Au(I) complex derived from **19·(PF**_**6**_**)**_**4**_ was also prepared.

**Scheme 5:**
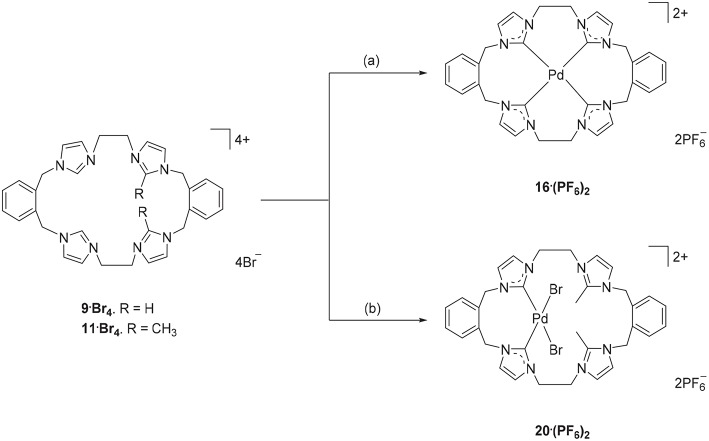
Synthesis of Pd(II) complex **16·(PF**_**6**_**)**_**2**_ and **20·(PF6)**_**2**_. (a) **9·Br**_**4**_, Ag_2_O (4.0 eq.), K_2_PdCl_4_, DMF, 85°C. (b) **11·Br**_**4**_, K_2_PdCl_4_, DMSO, 85°C, overnight.

Due to their potential to act as tetradentate ligands with metals that adopt square-planar coordination geometries, the Pd(II) complex of pro-ligand **9·Br**_**4**_ was prepared. A range of approached have been previously employed for the preparation of Pd(II)-NHC complexes, including *in situ* deprotonation and metallation (Baker et al., 2001; Fei et al., 2017) and transmetallation via an intermediate Ag(I) complex (Schulte to Brinke et al., 2013; Andrew et al., 2016). In this work the former *in situ* deprotonation and metallation approach was initially investigated by reacting **9·(PF**_**6**_**)**_**4**_ with Pd(OAc)_2_ in DMSO, however the desired Pd(II) complex could not be isolated. As described previously, a hexanuclear Ag(I) complex could be prepared from the pro-ligand **9·(PF**_**6**_**)**_**4**_ and in a second attempt to synthesize the Pd(II) complex, pro-ligand **9·Br**_**4**_ was first reacted with Ag_2_O to form the Ag(I) complex *in situ* followed by addition of K_2_PdCl_4_ (Scheme 5). ^1^H-NMR analysis of Pd(II) complex **16·(PF**_**6**_**)**_**2**_ showed a relatively simple spectrum with the C4/5 protons of the NHC groups resonating as two sets of doublets at 7.48 and 7.83 ppm. The benzylic protons resonate as two sets of doublet signals at 5.20 and 6.44 ppm (AX pattern), which is consistent with a rigid molecular structure in solution. Furthermore, the ^13^C-NMR spectrum showed a downfield shifted signal at 167.41 ppm, which corresponds to the NHC carbene carbon coordinated to the Pd(II) metal center.

The Ag(I) transmetallation approach did not successfully produced a Pd(II) complex of pro-ligand **11·Br**_**4**_. In this attempt, Ag_2_CO_3_ was used instead of Ag_2_O to avoid the undesirable oxidative cleavage of the C2-blocking methyl group which has been observed previously (Chianese et al., 2004). In a second synthetic attempt, the pro-ligand **11·Br**_**4**_ was reacted with K_2_PdCl_4_ in presence of NaOAc. The ^1^H-NMR of the crude product showed no peak for the C2-H proton for the “unblocked” imidazole units indicating that these groups are coordinated to the metal center. The C4/5 protons of the C2 “blocked” imidazolium group at 7.34 and 7.66 ppm suggesting that C2 blocked imidazolium unit did not react with the metal. Furthermore, the benzylic proton signals at 5.24 and 5.26 ppm and the ethylene linker signal at 4.61–4.70 ppm also indicated an asymmetrical structure of the title complex. The crystal structure in **Figure 5** also suggested the Pd(II) complex **20·(PF**_**6**_**)**_**2**_ was successfully synthesized. Unfortunately, it was unable to separate this complex as pure product.

### Structural Studies

**Figure 3:**
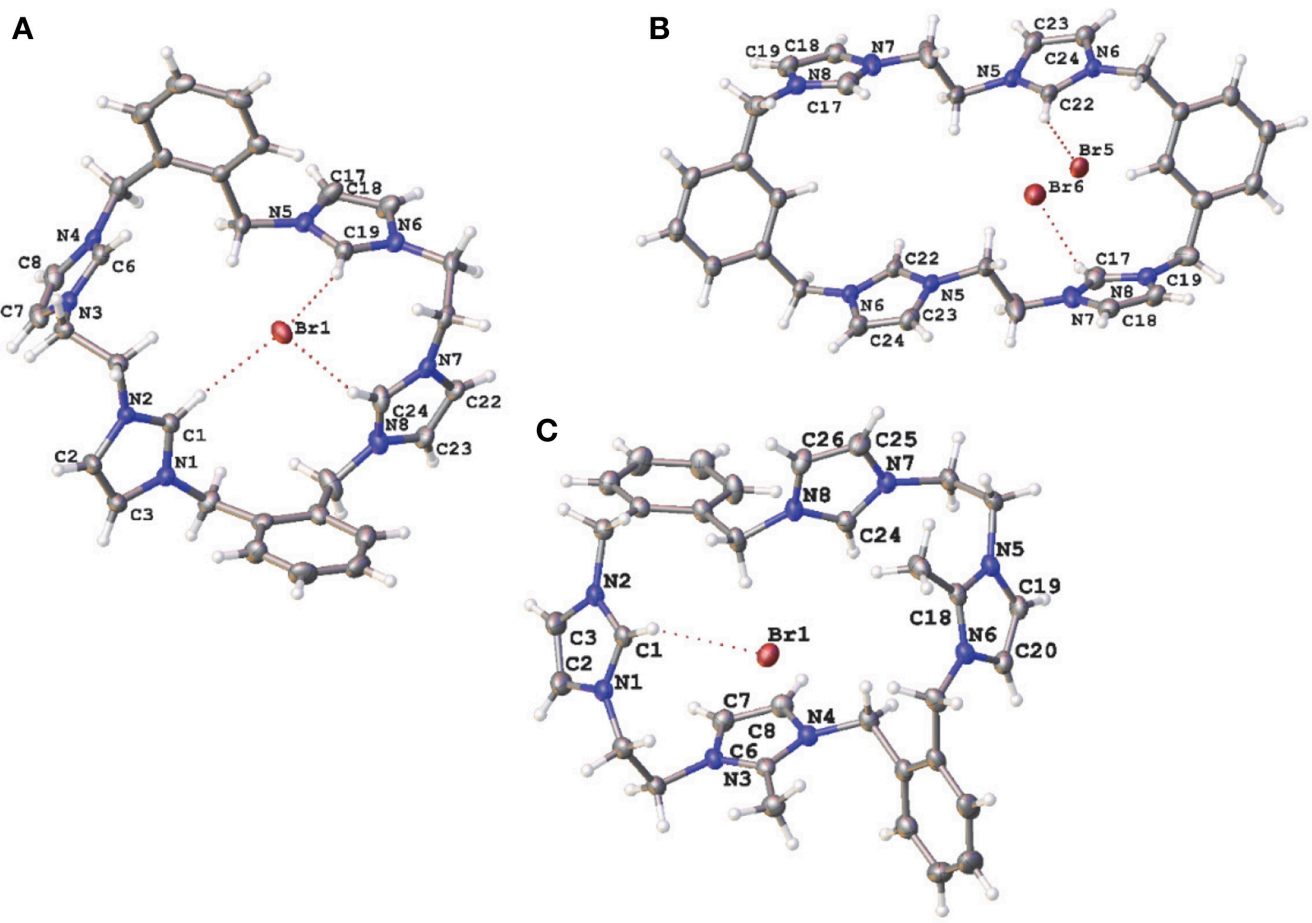
Representations of the X-ray crystal structures of **(A) 9·Br**_**4**_**, (B) 10·Br**_**3**_**PF**_**6**_, and **(C) 11·Br**_**4**_. Only counter ions involved in hydrogen bonding are included for clarity. Atomic displacement ellipsoids are shown at the 50% probability level.

Compounds **5·Br**_**2**_, **9·Br**_**4**_, **10·Br**_**3**_**PF**_**6**_, **11·Br**_**4**_, **15·Br**_**2**_**(PF**_**6**_**)**_**4**_, and **20·(PF**_**6**_**)**_**2**_ were characterized by X-ray crystallography. A representation of the precursor compound **5·Br**_**2**_ is shown in Figure S1 while representations of the *tetra*-imidazolium macrocycles **9·Br**_**4**_, **10·Br**_**3**_**PF**_**6**_, and **11·Br**_**4**_ are shown in Figure 3. In all cases these imidazolium salts display hydrogen bonding interactions between various hydrogens on the cationic imidazolium units and the bromide counter anions. For example, the shortest C2···Br distances for **5·Br**_**2**_, **9·Br**_**4**_, **10·Br**_**3**_**PF**_**6**_, and **11·Br**_**4**_, respectively, are: 3.4496(3); 3.42219(5) Å; 3.4822(4) Å; and 3.6552 (5) Å, respectively, which fall within a typical range of C-H···Br distance caused by hydrogen bonding (Yuan et al., 2002).

**Figure 4:**
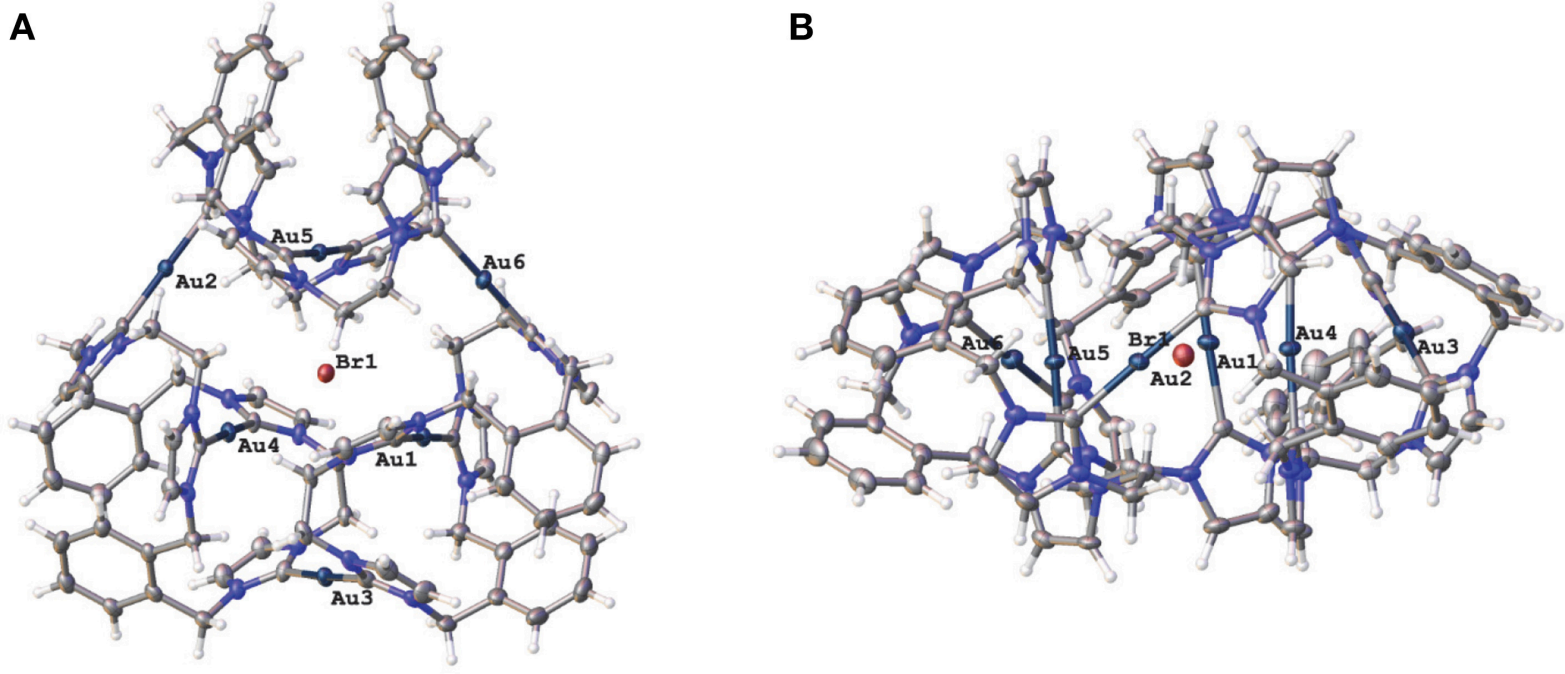
**(A)** Two representations of the X-ray crystal structures of **15·Br**_**2**_**(PF**_**6**_**)**_**4**_ **(A)** top view and **(B)** edge view. Hydrogen atoms are excluded and only the encapsulated bromide counterion included for clarity. Atomic displacement ellipsoids are shown at the 50% probability level.

Two representations of the X-ray crystal structure for the Au(I) complex **15·Br**_**2**_**(PF**_**6**_**)**_**4**_ are shown in Figure 4. This remarkable structure reveals that the Au(I) complex adopts a cyclic hexanuclear supramolecular assembly with the overall formula [Au_6_L_3_]^6+^. In the cation, each of the Au(I) centers adopt linear two-coordinate geometries, and they can be divided into two distinct groups of three atoms each. For the first group, each Au(I) atoms (Au1, Au4, and Au5) is bound to two NHC units on opposite sides of each of the three ligand molecules. While each of the Au(I) atoms in the second group (Au2, Au3, and Au6) are coordinated to NHC donors from adjacent ligand molecules, resulting in the formation of a metallo-macrocycle. In each case, the ligand molecules are bowl shaped to accommodate the two Au(I) coordination modes. Interestingly, the metallo-macrocycle is arrayed around an encapsulated central bromide counterion. This bromide ion displays interactions with Au(I) atoms Au1, Au4, and Au5 with the distances being 3.19488(5), 3.24731(5), and 3.23064(4) Å, respectively. Previously, a hexanuclear Ag(I) complex was reported for a *tetra*-carbene ligand linked by aliphatic butyl chains (Fei et al., 2017).

**Figure 5:**
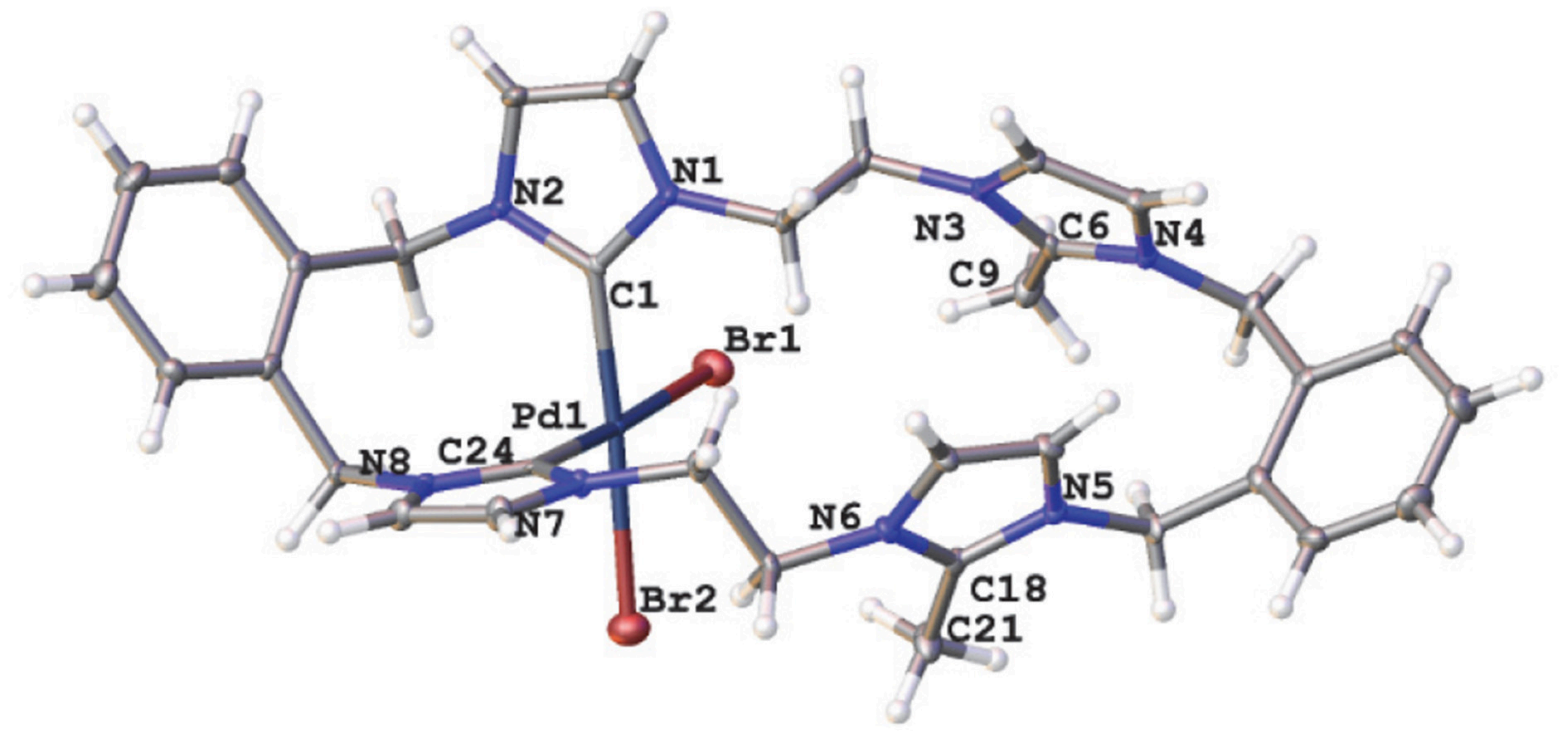
Representations of the X-ray crystal structures of the Pd(II) complex **20·(PF**_**6**_**)**_**2**_. Atomic displacement ellipsoids are shown at the 50% probability level.

A representation of the X-ray crystal structure for the Pd(II) **20·(PF**_**6**_**)**_**2**_ is shown in Figure 5. The molecular structure shows that the ligand is coordinated to the metal center through the “normal” NHC groups, with the C2 “blocked” groups present as cationic imidazolium units. The Pd(II) center is four-coordinate, with the two-remaining sited being occupied by bromide ions.

### Anion Binding Studies

Due to the tetracationic charge of the imidazolium linked macrocycles prepared in this work, the propensity of **9·(PF**_**6**_**)**_**4**_ and **10·(PF**_**6**_**)**_**4**_ to bind to the halide anions F^−^ Cl^−^, Br^−^, and I^−^ (as their *tetra*-n-butylammonium halide salts) was evaluated using ^1^H-NMR titration experiments. In initial studies, it was found that the addition of Bu_4_N·F to **10·(PF**_**6**_**)**_**4**_ in d_6_-DMSO caused an immediate color change to pale yellow. The ^1^H-NMR analysis showed that the color change occurred concurrently with a significant broadening and downfield shift of the imidazolium C2-H signal. In addition, the appearance of new unidentified ^1^H-NMR signals were observed which were consistent with decomposition of the macrocyclic receptor. As such this anion was not studied further.

**Figure 6:**
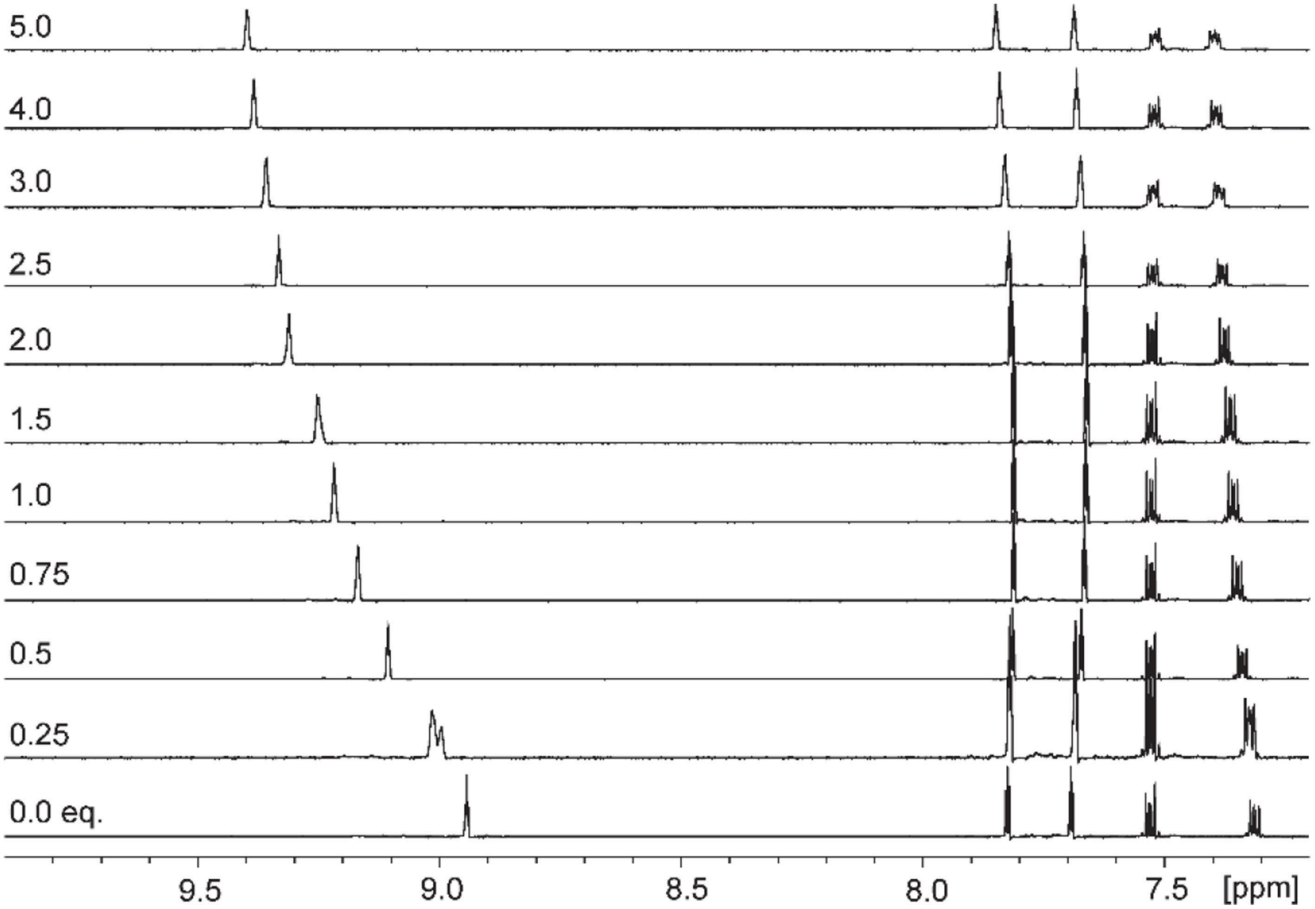
^1^H-NMR titration of **9·(PF**_**6**_**)**_**4**_ in d_6_-DMSO in the presence of increasing concentrations of Bu_4_N·Br.

**Figure 7:**
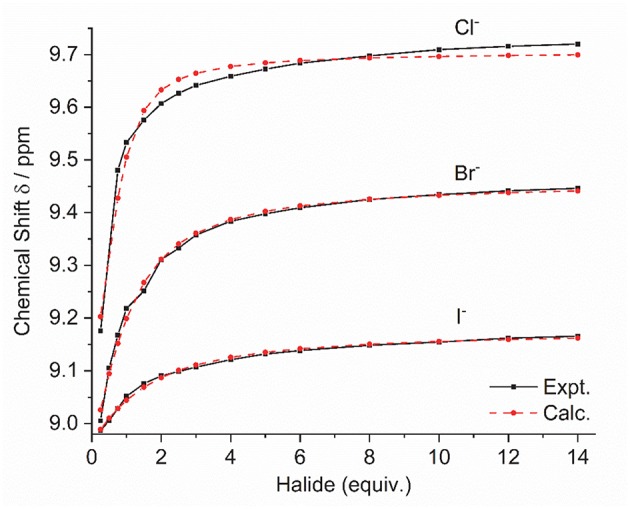
Experimental titration data (black line, squares) and fitted binding isotherms (red dashed line, circles) for the addition of the Bu_4_N^+^ halide anion to a solution of **9·(PF**_**6**_**)**_**4**_ in d_6_-DMSO.

Addition of increasing equivalents of Bu_4_N·Cl (0.25–14.0 eq.) to a solution of **9·(PF**_**6**_**)**_**4**_ in d_6_-DMSO caused a significant downfield shift of the resonance corresponding to the imidazolium C2-H signal from 8.96 to 9.64 ppm. In addition, the benzylic proton signal was also shifted downfield from 5.34 to 5.76 ppm. Unfortunately, this study was hampered by the gradual precipitation of the imidazolium salt at higher Cl^−^ concentrations. In a similar manner, addition of increasing equivalents of Bu_4_N·Br and Bu_4_N·I to **9·(PF**_**6**_**)**_**4**_ caused downfield shifts in the imidazolium C2-H signal, although to a lesser extent than that seen for Bu_4_N·Cl, however for these anions no precipitation of the macrocycle was seen. Figure 6 shows the ^1^H-NMR titration between **9·(PF**_**6**_**)**_**4**_ and Bu_4_N·Br, while Figure 7 shows the change in the imidazolium C2-H chemical shift for **9·(PF**_**6**_**)**_**4**_ in response to increasing equivalents of the added halide anion (solid black lines). Similar results were seen for the macrocyclic receptor **10·(PF**_**6**_**)**_**4**_ and the plot of experimental titration data is shown in Figure S2. Job plot analysis was then used to evaluate the stoichiometry of the interactions between the macrocyclic receptors and the anions Cl^−^, Br^−^, and I^−^. The results of these studies Figures S3, S4 showed that maxima occurred at close to χ = 0.5 indicating 1:1 stoichiometry for both receptor **9**^4+^ and **10**^4+^ with these anions.

Binding (association) constants *K*_a_ (M^−1^) for the *tetra*-imidazolium macrocycles **9·(PF**_**6**_**)**_**4**_ and **10·(PF**_**6**_**)**_**4**_ were determined by analysis of the ^1^H-NMR titration data using the computer program HypNMR 2018 (Frassineti et al., 1995, 2003). The fitted binding isotherms are shown in Figure 6 and Figure S2 (red dashed lines) and the calculated binding constants are given in Table 1. The values obtained for the compounds studied in this work are similar in magnitude to those reported previously for related compounds. For example, Beer et al. reported association constants of 420(23), 241(3), and 120(1) M^−1^ for a *tetra*-imidazolium macrocycle in d_6_-DMSO solution with the anions Cl^−^, Br^−^ and I^−^, respectively (Serpell et al., 2011).

**Table 1:** Association constants of the *tetra*-imidazolium macrocycles **9·(PF**_**6**_**)**_**4**_ and **10·(PF**_**6**_**)**_**4**_ with F^−^, Cl^−^, Br^−^, and I^−^.

	**9·(PF_**6**_)_**4**_**	**10·(PF_**6**_)_**4**_**
Cl^−^	501 (12)^a^	ppt^b^
Br^−^	126 (15)	63 (6)
I^−^	63 (6)	20 (2)

a *Precipitate forms after four equivalents of Bu_4_N·Cl added*;

b *precipitate forms rapidly and measurement of association constant not possible*.

## Conclusion

In conclusion, a novel stepwise synthetic strategy is reported that allows for the synthesis of both symmetrical and asymmetrical *tetra*-imidazolium linked macrocyclic compounds. The synthetic strategy is modular as initially a range of *bis*-bromoethylimidazolium bromide precursors were synthesized, which when combined with chosen *bis*(imidazolylmethyl)benzene molecules produced a range of *tetra*-imidazolium linked macrocycles. These *tetra*-imidazolium salts are of significant interest as they bind anions in solution and offer the potential for the development of sensors for biologically relevant anions. Using ^1^H-NMR titration studies, the association constants between **9·(PF**_**6**_**)**_**4**_ and **10·(PF**_**6**_**)**_**4**_ and the halide anions Cl^−^, Br^−^, and I^−^ were determined and these ranged between 501 and 20 M^−1^. Imidazolium salts are precursors for *N*-heterocyclic carbene (NHC) ligands and given that Ag(I) and Au(I) complexes of NHC ligands have been shown to display antimicrobial (Aweda et al., 2013; Shah et al., 2013) and anticancer(Barnard et al., 2004) properties, respectively, the Ag(I), Au(I), and Pd(II) NHC complexes derived from these *tetra*-imidazolium linked macrocycles were prepared. The Ag(I) complexes **14**^6+^ and the analogous Au(I) complex **15**^6+^ adopted intriguing hexanuclear structures with the general formula [M_6_L_3_]^6+^. Currently we are exploring the development of silver complexes of NHC ligands as potential antimicrobial agents and supramolecular assemblies such as complex **14**^6+^ are a particular focus for the slow release of silver ions and the results of these studies will be reported in due course.

## Experimental

### General Details

All solvents and chemicals were purchased from Sigma-Aldrich, Chem Supply, Alfa Aesar, and were used as received unless otherwise stated. Where necessary, solvents were further purified using an Innovative Technology Pure Solv solvent purification system. All experiments were performed under an atmosphere of N_2_ unless otherwise stated. ^1^H-NMR and ^13^C-NMR spectra were recorded on Bruker Advance 500 (500.023 MHz for ^1^H and 125.74 MHz for ^13^C) and spectra were referenced to solvent resonances. Where required, COZY, HSQC, HMBC, and NOESY 2-dimensional experiments were used to assist assignments. Mass spectra were obtained using an Agilent 6530 Q-TOF LC/MS mass spectrometer fitted with an Agilent electrospray ion (ESI) source.

### X-ray Crystallography

Single crystals suitable for X-ray diffraction studies were grown as follows: **5·Br**_**2**_ and **9·Br**_**4**_ diffusion of diethyl ether into an methanol solution of the title compound; **10·Br**_**3**_**PF**_**6**_ diffusion of diethyl ether into an acetonitrile solution of the title compound containing two drops of a solution of *tetra*-n-butylammonium bromide in acetonitrile; **11·Br**_**4**_ slow evaporation of a methanol solution of the title compound; **15·Br**_**2**_**(PF**_**6**_**)**_**4**_ diffusion of ethyl acetate into an acetonitrile solution of the title compound; **20·(PF**_**6**_**)**_**2**_ was grown by slow diffusion of diethyl ether into an acetonitrile solution of the titled compound. Crystallographic data for all structures determined are given in Table S1. For all samples, crystals were removed from the crystallization vial and immediately coated with paratone oil on a glass slide. A suitable crystal was mounted in Paratone oil on a glass fiber and cooled rapidly to 173 K in a stream of cold N_2_ using an Oxford low temperature device. Diffraction data were measured using an Rigaku Oxford Diffraction SuperNova X-ray Diffraction System mounted with Mo-Kα λ = 0.71073 Å and Cu-Kα λ = 1.54184. Data were reduced and corrected for absorption using the CrysAlis Pro program. The SHELXL2013-2 program was used to solve the structures with Direct Methods, with refinement by the Full-Matrix Least-Squares refinement techniques on F2. The non-hydrogen atoms were refined anisotropically and hydrogen atoms were placed geometrically and refined using the riding model. Coordinates and anisotropic thermal parameters of all non-hydrogen atoms were refined. All calculations were carried out using the program Olex2. Further XRD details are provided in the Supporting Information. CCDC 1885400-1885405 contains the supplementary crystallographic data for this paper. These data can be obtained free of charge from The Cambridge Crystallographic Data Centre via www.ccdc.cam.ac.uk/data_request/cif.

### ^1^H-NMR Titration Studies

A solution of the title compound (10 mg) in d_6_-DMSO (600 μL) and a 1.5 M solution of Bu_4_N·X (X = Cl, Br and I) in d_6_-DMSO were prepared, respectively. To the solution of title compound, increasing equivalents (0.25–14.0 eq.) of 1.5 M Bu_4_N·X solution was added and the resultant solution was thoroughly mixed. The ^1^H-NMR spectrum was recorded ~2 min after each addition at 302 K.

### Jobs Plot Analysis

A solution of the title compound (10 mg/mL) in d_6_-DMSO and a 0.050 M solution of Bu_4_N·X (X = Cl, Br and I) in d_6_-DMSO were prepared, respectively. A varied fraction of title compound solution and Bu_4_N·X solution was added and diluted with d_6_-DMSO to 600 μL to maintain the total concentration of substance at 10 mM. The resultant solution was thoroughly mixed and ^1^H-NMR spectrum was recorded at 302 K.

### Synthesis

**4**. Sodium hydride (1.14 g, 47.35 mmol) was added to a solution of 2-methylimidazole (3.11 g, 37.88 mmol) in DMF (50 mL) cooled to 0°C and the resultant mixture was stirred at RT for 1 h and α,α′-dibromo-*m*-xylene (5.00 g, 18.94 mmol) was then added. Stirring was continued at RT for 12 h and the mixture was then diluted with water (100 mL). The mixture was then extracted with CH_2_Cl_2_ (5 × 10 mL) and the combined organic extracts were washed with water (5 × 50 mL) and then brine (20 mL). The organic layer was dried with MgSO_4_ and the solvent was evaporated *in vacuo* yielding a yellow oil. Yield: 2.18 g, 43.2%. ^1^H-NMR (500.02 MHz, d_6_-DMSO): δ = 7.33 (t, ^3^*J*_H−H_ = 7.5 Hz, 1H, Ar*H*), 7.08 (d, ^3^*J*_H−H_ = 1.0 Hz, 2H, *H*_imi_), 7.02 (d, ^3^*J*_H−H_ = 8.0 Hz, 2H, Ar*H*), 6.96 (s, 1H, Ar*H*), 6.76 (d, ^3^*J*_H−H_ = 1.5 Hz, 2H, *H*_imi_), 5.12 (s, 4H, C*H*_2_), 2.19 (s, 6H, C*H*_3_). ^13^C-NMR (125.74 MHz, d_6_-DMSO): δ = 144.36 (*C*_q_), 138.55 (*C*_q_), 129.64 (*C*_Ar_), 126.96 (*C*_imi_), 126.59 (*C*_Ar_), 126.07 (*C*_Ar_), 120.70 (*C*_imi_), 48.94 (*C*H_2_), 13.18 (*C*H_3_). HRESI-MS^+^ (CH_3_CN): C_14_H_15_${\text{N}}_{4}^{+}$ m/z = 267.1543, calcd = 267.1604.

**5·Br**_**2**_. To a solution of 1,2-dibromoethane (21.69 mL, 251.7 mmol) in CH_3_CN (50 mL) stirred at 110°C, was added dropwise a solution of **1** (2.0 g, 8.39 mmol) in CH_3_CN (100 mL) over a period of 3 h. The resultant mixture was stirred at the same temperature for 12 h and then filtered whilst still hot. The filtrate was then evaporated *in vacuo* and the resulting solid was recrystallized from hot ethanol (20 mL) yielding a white crystalline solid. Yield: 2.19 g, 41.2%. ^1^H-NMR (500.02 MHz, d_6_-DMSO): δ = 9.50 (s, 2H, *H*_imi_), 7.98 (t, ^3^*J*_H−H_ = 1.8 Hz, 2H, *H*_imi_), 7.88 (t, ^3^*J*_H−H_ = 1.8 Hz, 2H, *H*_imi_), 7.49 (dd, ^3^*J*_H−H_ = 5.8 Hz, ^4^*J*_H−H_ = 3.5 Hz, 2H, Ar*H*), 7.30 (dd, ^3^*J*_H−H_ = 5.5 Hz, ^4^*J*_H−H_ = 3.5 Hz, 2H, Ar*H*), 5.75 (s, 4H, C*H*_2_), 4.69 (t, ^3^*J*_H−H_ = 6.0 Hz, 4H, C*H*_2_), 4.01 (t, ^3^*J*_H−H_ = 6.0 Hz, 4H, C*H*_2_). ^13^C-NMR (125.74 MHz, d_6_-DMSO): δ = 137.56 (*C*_imi_), 133.38 (*C*_q_), 130.10 (*C*_Ar_), 129.83 (*C*_Ar_), 123.42 (*C*_imi_), 123.38 (*C*_imi_), 50.76 (*C*H_2_), 49.61 (*C*H_2_), 32.90 (*C*H_2_). HRESI-MS^+^ (CH_3_OH): C_18_H_22_N_4_${\text{Br}}_{2}^{2+}$ m/z = 227.0094, calcd = 227.0090, C_18_H_22_N_4_${\text{Br}}_{3}^{+}$ m/z = 532.9367, calcd = 532.9369.

**6·Br**_**2**_. This compound was prepared using the same method as described for **5·Br**_**2**_ from 1,2-dibromoethane (10.80 mL, 125.90 mmol) and **2** (1.00 g, 4.20 mmol). The crude product was purified by a trituration with diethyl ether (3 × 10 mL) yielding a light brown oil. Yield: 0.62 g, 24.0%. ^1^H-NMR (500.02 MHz, d_6_-DMSO): δ = 9.63 (s, 2H, *H*_imi_), 7.95 – 7.97 (m, 4H, *H*_imi_), 7.65 (s, 1H, Ar*H*), 7.44 – 7.50 (m, 3H, Ar*H*), 5.56 (s, 4H, C*H*_2_), 4.69 (t, ^3^*J*_H−H_ = 5.9 Hz, 4H, C*H*_2_), 4.01 (t, ^3^*J*_H−H_ = 5.9 Hz, 4H, C*H*_2_). ^13^C-NMR (125.74 MHz, d_6_-DMSO): δ = 137.31 (*C*_imi_), 136.02 (*C*_q_), 130.19 (*C*_Ar_), 129.08 (*C*_Ar_), 128.90 (*C*_Ar_), 123.36 (*C*_imi_), 123.16 (*C*_imi_), 52.05 (*C*H_2_), 50.76 (*C*H_2_), 32.90 (*C*H_2_). HRESI-MS^+^ (CH_3_OH): C_18_H_22_N_4_${\text{Br}}_{2}^{2+}$ m/z = 227.0102, calcd = 227.0090.

**7·Br**_**2**_. This compound was using the same method as described for **5·Br**_**2**_ from 1,2-dibromoethane (4.85 mL, 56.31 mmol) and **3** (0.50 g, 1.88 mmol). The crude product was recrystallized from ethanol yielding a white solid. Yield: 0.62 g, 51.6%. ^1^H-NMR (500.02 MHz, d_6_-DMSO): δ = 7.90 (d, ^3^*J*_H−H_ = 2.1 Hz, 2H, *H*_imi_), 7.68 (d, ^3^*J*_H−H_ = 2.1 Hz, 2H, *H*_imi_), 7.40 (dd, ^3^*J*_H−H_ = 5.5 Hz, ^4^*J*_H−H_ = 3.4 Hz, 2H, Ar*H*), 6.82 (dd, ^3^*J*_H−H_ = 5.7 Hz, ^4^*J*_H−H_ = 3.5 Hz, 2H, Ar*H*), 5.67 (s, 4H, C*H*_2_), 4.68 (t, ^3^*J*_H−H_ = 5.9 Hz, 4H, C*H*_2_), 4.00 (t, ^3^*J*_H−H_ = 5.9 Hz, 4H, C*H*_2_), 2.69 (s, 6H, C*H*_3_). ^13^C-NMR (125.74 MHz, d_6_-DMSO): δ = 146.15 (*C*_q_), 132.62 (*C*_q_), 129.28 (*C*_Ar_), 126.95 (*C*_Ar_), 122.65 (*C*_imi_), 122.43 (*C*_imi_), 49.29 (*C*H_2_), 48.85 (*C*H_2_), 31.96 (*C*H_2_), 10.70 (*C*H_3_). HRESI-MS^+^ (CH_3_OH): C_20_H_26_N_4_${\text{Br}}_{2}^{2+}$ m/z = 241.0306, calcd = 241.0246.

**8·Br**_**2**_. This compound was prepared using the same method as described for **5·Br**_**2**_ from 1,2-dibromoethane (8.15 mL, 94.61 mmol) and **4** (0.84 g, 3.15 mmol). The crude product was recrystallized from ethanol yielding a white solid. Yield: 0.76 g, 37.5%. ^1^H-NMR (500.02 MHz, d_6_-DMSO): δ = 7.81 (s, 2H, *H*_imi_), 7.79 (s, 2H, *H*_imi_), 7.46 (t, ^3^*J*_H−H_ = 7.8 Hz, 1H, Ar*H*), 7.33 (s, 1H, Ar*H*), 7.27 (d, ^3^*J*_H−H_ = 7.7 Hz, 2H, Ar*H*), 5.47 (s, 4H, C*H*_2_), 4.62 (t, ^3^*J*_H−H_ = 6.0 Hz, 4H, C*H*_2_), 3.93 (t, ^3^*J*_H−H_ = 5.9 Hz, 4H, C*H*_2_), 2.67 (s, 6H, C*H*_3_). ^13^C-NMR (125.74 MHz, d_6_-DMSO): δ = 145.47 (*C*_q_), 135.76 (*C*_q_), 130.32 (*C*_Ar_), 128.15 (*C*_Ar_), 127.59 (*C*_Ar_), 122.40 (*C*_imi_), 122.35 (*C*_imi_), 50.88 (*C*H_2_), 49.17 (*C*H_2_), 31.78 (*C*H_2_), 10.37 (*C*H_3_). HRESI-MS^+^ (CH_3_OH): C_20_H_26_N_4_${\text{Br}}_{2}^{2+}$ m/z = 241.0294, calcd = 241.0246, HRESI-MS^+^ (CH_3_OH): C_20_H_26_N_4_${\text{Br}}_{3}^{+}$ m/z = 560.9702, calcd = 560.9682.

**9·Br**_**4**_. Solutions of **1** (0.31 g, 1.30 mmol) in CH_3_CN (100 mL) and **5·Br**_**2**_ (0.80 g, 1.30 mmol) in DMF (100 mL) were added simultaneously dropwise to 150 mL of CH_3_CN heated at 110°C over a period of 3 h. The mixture was then stirred at the same temperature for a further 5 d during which time a precipitate formed. The precipitate was collected and washed with CH_3_CN (3 × 5 mL) and recrystallized from a mixture of methanol and isopropanol yielding a white crystalline solid. Yield: 0.33 g, 29.7%. ^1^H-NMR (500.02 MHz, d_6_-DMSO): δ = 9.45 (s, 4H, *H*_imi_), 7.90 (t, ^3^*J*_H−H_ = 1.5 Hz, 4H, *H*_imi_), 7.77 (t, ^3^*J*_H−H_ = 1.5 Hz, 4H, *H*_imi_), 7.52 (dd, ^3^*J*_H−H_ = 5.8 Hz, ^4^*J*_H−H_ = 3.0 Hz, 4H, Ar*H*), 7.40 (dd, ^3^*J*_H−H_ = 5.5 Hz, ^4^*J*_H−H_ = 3.5 Hz, 4H, Ar*H*), 5.68 (s, 8H, C*H*_2_), 4.81 (s, 8H, C*H*_2_). ^13^C-NMR (125.74 MHz, d_6_-DMSO): δ = 136.75 (C_imi_), 133.13 (C_q_), 131.04 (C_Ar_), 130.38 (C_Ar_), 123.66 (C_imi_), 123.51(C_imi_), 49.95 (*C*H_2_). HRESI-MS^+^ (CH_3_CN): C_32_H_36_${\text{N}}_{8}^{4+}$ m/z = 133.0655, calcd = 133.0760, C_32_H_36_N_8_P_3_${\text{F}}_{18}^{+}$ m/z = 967.2020, calcd = 967.1967.

**10·Br**_**4**_. This compound was prepared using the same method as described for **9·Br**_**4**_. from **2** (0.24 g, 1.01 mmol) and **6·Br**_**2**_ (0.62 g, 1.01 mmol). Yield: 0.16 g, 18.0%. ^1^H-NMR (500.02 MHz, d_6_-DMSO): δ = 9.53 (s, 4H, *H*_imi_) 7.83 (t, ^3^*J*_H−H_ = 1.7 Hz, 4H, *H*_imi_), 7.80 (t, ^3^*J*_H−H_ = 1.7 Hz, 4H, *H*_imi_), 7.51 (s, 2H, Ar*H*), 7.36 (t, ^3^*J*_H−H_ = 7.7 Hz, 4H, Ar*H*), 7.26–7.28 (m, 4H, Ar*H*), 5.45 (s, 8H, C*H*_2_), 4.84 (s, 8H, C*H*_2_). ^13^C-NMR (125.74 MHz, d_6_-DMSO): δ = 137.50 (*C*_imi_), 135.80 (*C*_q_), 130.70 (*C*_Ar_), 128.92 (*C*_Ar_), 128.87 (*C*_Ar_), 128.39 (*C*_imi_), 52.21 (*C*H_2_), 48.83 (*C*H_2_). HRESI-MS^+^ (CH_3_CN): C_32_H_36_${\text{N}}_{8}^{4+}$ m/z = 133.0686, C_32_H_36_${\text{N}}_{8}^{4+}$ calcd = 133.0760, C_32_H_36_N_8_P_2_${\text{F}}_{12}^{2+}$ m/z = 411.1099, C_32_H_36_N_8_P_2_${\text{F}}_{12}^{2+}$ calcd = 411.1163, C_32_H_36_N_8_P_3_${\text{F}}_{18}^{+}$ m/z = 967.1990, C_32_H_36_N_8_P_3_${\text{F}}_{18}^{+}$ calcd = 967.1967.

**11·Br**_**4**_. Solutions of **1** (0.19 g, 0.80 mmol) in CH_3_CN (50 mL) and **7·Br**_**2**_ (0.50 g, 0.80 mmol) in DMF (50 mL) were added simultaneously dropwise to a solution of Bu_4_N·Br (1.55 g, 4.80 mmol) in CH_3_CN (150 mL) heated at 110°C over a period of 3 h. The mixture was stirred at the same temperature for a further 5 d during which time a precipitate formed. The precipitate was collected and washed with CH_3_CN (3 × 5 mL) and then recrystallized from a mixture of methanol and diethyl ether yielding a white crystalline solid. Yield: 0.10g, 7.5%. ^1^H-NMR (500.02 MHz, d_6_-DMSO): δ = 9.76 (s, 2H, *H*_imi_), 8.00 (s, 2H, *H*_imi_), 7.65 (s, 2H, *H*_imi_), 7.49–7.53 (m, 10H, Ar*H, H*_imi_), 7.23 (dd, ^3^*J*_H−H_ = 5.4 Hz, ^4^*J*_H−H_ = 3.6 Hz, 4H, Ar*H*), 5.52 (s, 4H, C*H*_2_), 5.47 (s, 4H, C*H*_2_), 4.81 – 4.83 (m, 4H, C*H*_2_), 4.70 – 4.72 (m, 4H, C*H*_2_), 2.64 (s, 6H, C*H*_3_). ^13^C-NMR (125.74 MHz, d_6_-DMSO): δ = 145.98 (*C*_q_), 137.44 (*C*_imi_), 132.92 (*C*_q_), 132.34 (*C*_q_), 131.15 (*C*_Ar_), 130.47 (*C*_Ar_), 129.97 (*C*_Ar_), 129.56 (*C*_Ar_), 123.98 (*C*_imi_), 123.42 (*C*_imi_), 122.83 (*C*_imi_), 122.67 (*C*_imi_), 49.48 (*C*H_2_), 49.38 (*C*H_2_), 49.19 (*C*H_2_), 47.90 (*C*H_2_), 10.02 (*C*H_3_). HRESI-MS^+^ (CH_3_CN): C_32_H_36_N_8_P_2_${\text{F}}_{12}^{2+}$ m/z = 425.1335, calcd = 425.1324, C_34_H_40_N_8_P_3_${\text{F}}_{18}^{+}$ m/z = 995.2323, calcd 995.2296.

**12·Br**_**4**_. This compound was prepared using the same method described for **11·Br**_**4**_ from **2** (0.11g, 0.47 mmol) and **8·Br**_**2**_ (0.30 g, 0.47 mmol). The crude product was recrystallized from methanol yielding a white crystalline solid. Yield: 0.068 g, 16.5%. ^1^H-NMR (500.023 MHz, d_6_-DMSO): δ = 9.53 (s, 2H, *H*_imi_), 7.80-7.84 (m, 4H, *H*_imi_), 7.62 (d, ^3^*J*_H−H_ = 2.2 Hz, 2H, *H*_imi_), 7.48 (d, ^3^*J*_H−H_ = 2.2 Hz, 2H, *H*_imi_), 7.46 (s, 1H, Ar*H*), 7.40 (t, ^3^*J*_H−H_ = 7.7 Hz, 1H, Ar*H*), 7.34 (s, 1H, Ar*H*), 7.24 – 7.30 (m, 3H, Ar*H*), 7.12 (dd, ^3^*J*_H−H_ = 7.7 Hz, ^4^*J*_H−H_ = 1.3 Hz, 2H, Ar*H*), 5.42 (s, 4H, C*H*_2_), 5.38 (s, 4H, C*H*_2_), 4.74 (s, 8H, C*H*_2_), 2.63 (s, 6H, C*H*_3_). ^13^C-NMR (125.74 MHz, d_6_-DMSO): δ = 145.85 (*C*_q_), 137.61 (*C*_imi_), 135.54 (*C*_q_), 135.39 (*C*_q_), 128.47 (*C*_Ar_), 128.04 (*C*_Ar_), 123.50 (*C*_imi_), 123.41 (*C*_imi_), 122.30 (*C*_imi_), 122.23 (*C*_imi_), 52.19 (*C*H_2_), 50.92 (*C*H_2_), 48.33 (*C*H_2_), 47.73 (*C*H_2_), 10.49 (*C*H_3_). HRESI-MS^+^ (CH_3_CN): C_32_H_36_N_8_${\text{Br}}_{2}^{2+}$ m/z = 360.0861, calcd = 360.0865.

**13·(PF**_**6**_**)**_**4**_ This compound was prepared using the same method described for **11·Br**_**4**_ from **2** and **5·Br**_**2**_ (0.52 g, 0.85 mmol) and Bu_4_N·Br (1.37 g, 4.25 mmol). The crude product was then dried *in vacuo* and re-dissolved in water (5 mL) and then filtered through a plug of celite. To this solution, a solution of KPF_6_ saturated in aqueous (3 mL) was added to obtain a white precipitate. The precipitate was washed with isopropanol (5 mL) and recrystallized by vapor diffusion of CH_3_CN/diethyl ether to obtain a white crystalline. Yield: 0.15 g, 16.0%. ^1^H-NMR (500.023 MHz, d_6_-DMSO): δ = 9.13 (s, 2H, *H*_imi_), 8.99 (s, 2H, *H*_imi_), 7.84 (t, ^3^*J*_H−H_ = 1.8 Hz, 2H, *H*_imi_), 7.72 (t, ^3^*J*_H−H_ = 1.8 Hz, 2H, *H*_imi_), 7.71 (t, ^3^*J*_H−H_ = 1.8 Hz, 2H, *H*_imi_), 7.65 (t, ^3^*J*_H−H_ = 1.8 Hz, 2H, *H*_imi_), 7.39 – 7.43 (m, 3H, Ar*H*), 7.11 (s, 1H, Ar*H*), 7.02 (dd, ^3^*J*_H−H_ = 7.7 Hz, ^4^*J*_H−H_ = 1.1 Hz, 2H, Ar*H*), 6.92 – 6.96 (m, 2H, Ar*H*), 5.37 (s, 4H, C*H*_2_), 5.36 (s, 4H, C*H*_2_), 4.72–4.80 (m, 8H, C*H*_2_). ^13^C-NMR (125.74 MHz, d_6_-DMSO): δ = 137.40 (*C*_imi_), 137.28 (*C*_imi_), 135.56 (*C*_q_), 132.55 (*C*_q_), 130.16 (*C*_Ar_), 130.04 (*C*_Ar_), 129.30 (*C*_Ar_), 127.75 (*C*_Ar_), 127.35(*C*_Ar_), 123.99 (*C*_imi_), 123.74 (*C*_imi_), 123.72 (*C*_imi_), 123.57 (*C*_imi_), 52.14 (*C*H_2_), 49.50 (*C*H_2_), 49.20 (*C*H_2_). HRESI-MS^+^ (CH_3_CN): C_32_H_36_${\text{N}}_{8}^{4+}$ m/z = 133.0750, calcd = 133.0760, C_32_H_36_N_8_P_2_${\text{F}}_{12}^{2+}$ m/z = 411.1158, calcd = 411.1168, C_32_H_36_N_8_P_3_${\text{F}}_{18}^{+}$ m/z = 967.1985, calcd = 967.1983.

**14·(PF**_**6**_**)**_**6**_. A slurry of **9·Br**_**4**_ (0.10 g, 0.12 mmol) and Ag_2_O (0.11 g, 0.47 mmol) in DMF (10 mL) was stirred at 50°C for 3 d with the exclusion of light. Diethyl ether (~50 mL) was then added to the mixture and a gray precipitate formed which was collected and dissolved in hot water (5 mL). The solution was clarified by filtration through syringe filter and a saturated solution of KPF_6_ (2 mL) was added to obtain an off-white precipitate. The solid collected and washed with hot isopropanol (2 mL) and then recrystallized from a mixture of CH_3_CN and diethyl ether to obtain a white crystalline solid. Yield: 0.026 g, 21.4%. ^1^H-NMR (500.02 MHz, d_6_-DMSO): δ = 7.70 (s, 6H, *H*_imi_), 7.68 (s, 6H, *H*_imi_), 7.52 (d, ^3^*J*_H−H_ = 7.5 Hz, 6H, Ar*H*), 7.17 (t_app_, *J* = 7.5 Hz, 6H, Ar*H*,), 7.09 (s, 6H, *H*_imi_), 6.94 (t_app_, *J* = 7.5 Hz, 6H, Ar*H*), 6.76 (s, 6H, *H*_imi_), 5.75 (d, ^2^*J*_H−H_ = 15.0 Hz, 12H, C*H*_2_), 5.68 (d, ^3^*J*_H−H_ = 7.5 Hz, 6H, Ar*H*), 5.60 (d, ^2^*J*_H−H_ = 17.0 Hz, 6H, C*H*_2_), 4.98 (d, ^2^*J*_H−H_ = 14.5 Hz, 6H, C*H*_2_), 4.65 (t, ^3^*J*_H−H_ = 12.5 Hz, 6H, C*H*_2_), 4.52 (t, ^3^*J*_H−H_ = 12.5 Hz, 6H, C*H*_2_), 4.21 (d, ^3^*J*_H−H_ = 12.0 Hz, 6H, C*H*_2_), 2.48 (s, 6H, C*H*_2_). ^13^C-NMR (125.74 MHz, d_6_-DMSO): δ = 186.31 (d, ^1^*J* = 182.32 Hz, ^107^Ag-*C*_carbene_), 186.31 (d, ^1^*J* = 209.99 Hz, ^109^Ag-*C*_carbene_), 178.75 (d, ^1^*J* = 182.32 Hz, ^107^Ag-*C*_carbene_), 178.75 (d, ^1^*J* = 209.99 Hz, ^109^Ag-*C*_carbene_), 135.80 (*C*_q_), 131.60 (*C*_q_), 130.88 (*C*_Ar_), 129.41 (*C*_Ar_), 128.14 (*C*_Ar_), 124.63 (*C*_Ar_), 123.90 (*C*_imi_), 123.78(*C*_imi_), 121.75 (*C*_imi_), 55.37 (*C*H_2_), 52.58 (*C*H_2_), 51.66 (*C*H_2_), 51.24 (*C*H_2_), 50.50 (*C*H_2_). HRESI-MS^+^ (CH_3_CN): C_96_H_96_N_24_${\text{Ag}}_{6}^{6+}$ m/z = 372.0334, calcd = 372.0419, C_96_H_96_N_24_Ag_6_P_3_${\text{F}}_{18}^{3+}$ m/z = 889.0490, calcd = 889.0485.

**15·Br**_**6**_. A slurry of **9·Br**_**4**_ (0.10 g, 0.12 mmol) and (THT)AuCl (0.083 g, 0.26 mmol) in DMF (10 mL) was stirred at 110°C for 0.5 h. To this mixture NaOAc (0.050 g, 0.59 mmol) was added and the solution was stirred at the same temperature for 1 h. The resultant mixture was cooled to RT and then diethyl ether (50 mL) was added to obtain a white precipitate. The precipitate was collected and recrystallized from hot methanol (~5 mL). Yield: 0.048 g, 54.2%. ^1^H-NMR (500.02 MHz, d_6_-DMSO): δ = 7.83 (s, 6H, *H*_imi_), 7.72 (s, 6H, *H*_imi_), 7.57 (d, ^3^*J*_H−H_ = 7.5 Hz, 6H, Ar*H*), 7.21 (t_app_, *J* = 7.5 Hz, 6H, Ar*H*) 7.11 (s, 6H, *H*_imi_), 6.93 (t_app_, *J* = 7.5 Hz, 6H, Ar*H*) 6.84 (s, 6H, *H*_imi_), 6.07 (d, ^2^*J*_H−H_ = 16.5 Hz, 6H, C*H*_2_), 5.74 – 5.78 (m, 12H, Ar*H*, C*H*_2_), 5.66 (d, ^3^*J*_H−H_ = 16.5 Hz, 6H, C*H*_2_), 5.06 (d, ^3^*J*_H−H_ = 14.0 Hz, 12H, C*H*_2_), 4.49 (t, ^3^*J*_H−H_ = 12.8 Hz, 6H, C*H*_2_), 4.37 (d, ^3^*J*_H−H_ = 12.5 Hz, 6H, C*H*_2_), 2.33 (d, ^3^*J*_H−H_ = 12.5 Hz, 6H, C*H*_2_). ^13^C-NMR (125.74 MHz, d_6_-DMSO): δ = 186.31 (*C*_carbene_), 181.48 (*C*_carbene_), 135.84 (*C*_q_), 131.37 (*C*_q_), 131.02 (*C*_Ar_), 129.62 (*C*_Ar_), 127.99 (*C*_Ar_), 124.59 (*C*_Ar_), 123.95 (*C*_imi_), 123.64 (*C*_imi_), 121.86 (*C*_imi_), 51.89 (*C*H_2_), 51.27 (*C*H_2_), 50.22 (*C*H_2_). HRESI-MS^+^ (CH_3_CN): C_96_H_96_N_24_${\text{Au}}_{6}^{6+}$ m/z = 461.1022, calcd = 461.1035.

**16·(PF**_**6**_**)**_**2**_. A slurry of **9·Br**_**4**_ (0.050 g, 0.059 mmol) and Ag_2_O (0.030 g, 0.13 mmol) in DMF (5 mL) was stirred at 50°C for 12 h with the exclusion of light. To this mixture K_2_PdCl_4_ (0.019 g, 0.059 mmol) was added and stirring was continued for a further stirred for 12 h at 80°C. The reaction mixture was clarified by centrifugation and diethyl ether (30 mL) was added to the supernatant yielding a gray precipitate. The precipitate was collected and re-dissolved in hot water (5 mL) and the solution filtered through a syringe filter. To the filtrate, a saturated solution of KPF_6_ (2 mL) was added to obtain an off-white precipitate. The precipitate was dried *in vacuo* and then recrystallized from a mixture of CH_3_CN and diethyl ether to obtain a white crystalline solid. Yield: 0.0052 g, 9.6%. ^1^H-NMR (500.02 MHz, d_6_-DMSO): δ = 7.87 – 7.92 (m, 4H, Ar*H*), 7.83 (d, ^3^*J*_H−H_ = 2.0 Hz, 4H, *H*_imi_), 7.48 (d, ^3^*J*_H−H_ = 2.0 Hz, 4H, *H*_imi_), 7.42 – 7.47 (m, 4H, Ar*H*), 6.44 (d, ^3^*J*_H−H_ = 15.0 Hz, 4H, C*H*_2_), 5.20 (d, ^3^*J*_H−H_ = 14.7 Hz, 4H, C*H*_2_), 4.98 – 5.06 (m, 4H, C*H*_2_), 4.54 – 4.61 (m, 4H, C*H*_2_). ^13^C-NMR (125.74 MHz, d_6_-DMSO): δ = 167.41 (*C*_carbene_), 135.56 (*C*_q_), 131.73 (*C*_Ar_), 129.83 (*C*_Ar_), 124.96 (*C*_imi_), 122.68 (*C*_imi_), 50.79 (*C*H_2_), 47.39 (*C*H_2_). HRESI-MS^+^ (CH_3_CN): C_32_H_32_N_8_Pd^2+^ m/z = 317.0889, calcd = 317.0887.

**17·(PF**_**6**_**)**_**3**_. This compound was prepared using the same method as described for **14·(PF**_**6**_**)**_**6**_ from **10·Br**_**4**_ (0.050 g, 0.059 mmol) and Ag_2_O (0.057 g, 0.24 mmol). Yield: 0.0050 g, 6.2%. ^1^H-NMR (500.02 MHz, CD_3_CN): δ = 8.37 (t, *J* = 1.5 Hz, 2H, *H*_imi_), 7.45 – 7.53 (m, 12H, Ar*H, H*_imi_), 7.21 (t, ^3^*J*_H−H_ = 1.8 Hz, 2H, *H*_imi_), 6.98 (s, 2H, Ar*H*), 6.95 (t, ^3^*J*_H−H_ = 1.9 Hz, 2H, *H*_imi_) 5.31 (s, 4H, C*H*_2_), 5.08 (s, 4H, C*H*_2_), 4.60 (d, ^3^*J*_H−H_ = 4.3 Hz, 4H, C*H*_2_), 4.55 (d, ^3^*J*_H−H_ = 4.7 Hz, 4H, C*H*_2_). ^13^C-NMR (125.74 MHz, CD_3_CN): δ = 180.39 (d, ^1^*J* = 183.58 Hz, ^107^Ag-*C*_carbene_), 180.39 (d, ^1^*J* = 211.24 Hz, ^109^Ag-*C*_carbene_), 138.62 (*C*_q_), 136.79 (*C*_q_), 136.18 (*C*_imi_), 130.86 (*C*_Ar_), 129.82 (*C*_imi_), 129.48 (*C*_imi_), 126.74 (*C*_Ar_), 125.47 (*C*_Ar_), 125.42 (*C*_Ar_), 124.47 (*C*_imi_), 124.12 (*C*_imi_), 122.39 (*C*_Ar_), 122.35 (*C*_Ar_), 55.33 (*C*H_2_), 53.45 (*C*H_2_), 52.73 (*C*H_2_), 50.96 (*C*H_2_). HRESI-MS^+^ (CH_3_CN): C_32_H_34_N_8_Ag^3+^ m/z = 213.0643, calcd = 213.0646, C_32_H_34_N_8_${\text{AgPF}}_{6}^{2+}$ m/z = 391.2845, calcd = 391.0794, C_32_H_34_N_8_AgP_2_${\text{F}}_{12}^{+}$ m/z = 927.1228, calcd = 927.1235.

**18·(PF**_**6**_**)**_**2**_. This compound was prepared using the same method as described for **15·Br**_**6**_ from **11·Br**_**4**_ (0.050 g, 0.057 mmol), (THT)AuCl (0.040 g, 0.13 mmol), and NaOAc (0.019 g, 0.23 mmol). For exchange of the bromide anion to hexafluorophosphate the crude product was dissolved in water (3 mL) and the solution filtered through celite. To this solution, a saturated solution of KPF_6_ (2 mL) was added to obtain a white precipitate which was then recrystallized from a mixture of CH_3_CN and diethyl ether yielding a white crystalline solid. Yield: 0.020 g, 29.9%. ^1^H-NMR (500.02 MHz, CD_3_CN): δ = 7.55 – 7.57 (m, 1H, Ar*H*), 7.46 – 7.48 (m, 1H, Ar*H*), 7.44 (d, ^3^*J*_H−H_ = 2.0 Hz, 1H, *H*_imi_), 7.41−7.42 (m, 1H, Ar*H*), 7.38 (s, 1H, *H*_imi_), 7.23 (d, ^3^*J*_H−H_ = 2.1 Hz, 1H, *H*_imi_), 7.21-7.22 (m, 1H, Ar*H*), 7.15 (d, ^3^*J*_H−H_ = 1.9 Hz, 1H, *H*_imi_), 5.53 (s, 2H, C*H*_2_), 5.49 (s, 2H, C*H*_2_), 4.66 – 4.67 (m, 2H, C*H*_2_), 4.57 – 4.59 (m, 2H, C*H*_2_), 2.54 (s, 3H, C*H*_3_). ^13^C-NMR (125.74 MHz, CD_3_CN): δ = 145.20 (*C*_carbene_), 133.61 (*C*_q_), 130.51 (*C*_Ar_), 130.43 (*C*_Ar_), 130.22 (*C*_Ar_), 129.75 (*C*_Ar_), 122.74 (*C*_imi_), 122.50 (*C*_imi_), 122.24 (*C*_imi_), 122.05 (*C*_imi_), 51.84 (*C*H_2_), 50.05 (*C*H_2_), 49.69 (*C*H_2_), 48.48 (*C*H_2_), 10.36 (*C*H_3_). HRESI-MS^+^ (CH_3_CN): C_34_H_38_N_8_Au_2_${\text{Br}}_{2}^{2+}$ m/z = 556.0369, calcd = 556.0443.

**19·(PF**_**6**_**)**_**2**_. This compound was prepared using the same method as that described for **15·Br**_**6**_ from **11·Br**_**4**_ (0.030 g, 0.034 mmol), (THT)AuCl (0.024 g, 0.075 mmol), and NaOAc (0.11 g, 0.14 mmol). For exchange of the bromide anion to hexafluorophosphate the crude product was dissolved in water (3 mL) and the solution filtered through celite. To this solution, a saturated solution of KPF_6_ (2 mL) was added to obtain a white precipitate which was then recrystallized from a mixture of CH_3_CN and diethyl ether yielding a white crystalline solid. Yield: 0.0070 g, 1.5%. ^1^H-NMR (500.02 MHz, d_6_-DMSO): δ = 7.71 (d, ^3^*J*_H−H_ = 1.9 Hz, 2H, *H*_imi_), 7.62 (d, ^3^*J*_H−H_ = 1.9 Hz, 2H, *H*_imi_), 7.56 (d, ^3^*J*_H−H_ = 1.9 Hz, 2H, *H*_imi_), 7.39−7.43 (m, 3H, Ar*H, H*_imi_), 7.28 – 7.38 (m, 3H, Ar*H*), 7.14 – 7.19 (m, 1 H, Ar*H*), 7.07 – 7.14 (m, 1H, Ar*H*), 6.56 – 6.69 (m, 2H, Ar*H*), 5.31 (s, 4H, C*H*_2_), 5.06 (s, 4H, C*H*_2_), 4.67 (s, 4H, C*H*_2_), 4.66 (s, 4H, C*H*_2_), 2.40 (s, 6H, C*H*_3_). ^13^C-NMR (125.74 MHz, d_6_-DMSO): δ = 172.52 (*C*_carbene_), 144.61 (*C*_q_), 136.79 (*C*_q_), 134.11 (*C*_q_), 130.12 (*C*_Ar_), 127.96 (*C*_Ar_), 125.29 (*C*_Ar_), 123.45 (*C*_imi_), 122.28 (*C*_imi_), 121.98 (*C*_imi_), 121.91 (*C*_imi_), 52.82 (*C*H_2_), 50.44 (*C*H_2_), 49.84 (*C*H_2_), 48.11 (*C*H_2_), 9.19 (*C*H_3_). HRESI-MS^+^ (CH_3_CN): C_34_H_38_N_8_Au_2_${\text{Br}}_{2}^{2+}$ m/z = 556.0422, calcd = 556.0443.

**20·(PF**_**6**_**)**_**2**_. To a solution of **11·Br**_**4**_ (0.050 g, 0.057 mmol) and K_2_PdCl_4_ (0.019 g, 0.057 mmol) in DMSO (5 mL) at 85°C, NaOAc (0.019 g, 0.23 mmol) was added and the reaction mixture was stirred same temperature for 12 h. The resultant mixture was then diluted with acetone (5 mL) followed by adding diethyl ether (20 mL) to form a precipitate. The crude precipitate was collected and then re-dissolved in water and filtered through a plug of Celite followed by addition of a saturated solution of KPF_6_ (3 mL). The resultant precipitate was collected and washed with isopropanol (5 mL) and diethyl ether (2 × 5 mL) and then recrystallized from a mixture of CH_3_CN and diethyl ether yielding the product as a pale yellow solid. NMR analysis showed this material to be impure and the ^1^H-NMR spectrum is reported for the impure material. ^1^H-NMR (500.02 MHz, CD_3_CN): δ = 7.66 (s, 2H, *H*_imi_), 7.55 – 7.57 (m, 4H, Ar*H*), 7.35−7.40 (m, 2H, Ar*H*), 7.34 (s, 2H, *H*_imi_), 7.22 – 7.24 (m, 2H, *H*_imi_), 7.03 (s, 2H, *H*_imi_), 7.02 (s, 2H, *H*_imi_), 5.26 (s, 4H, C*H*_2_), 5.24 (s, 4H, C*H*_2_), 4.61 – 4.70 (m, 8H, C*H*_2_), 2.57 (s, 6H, C*H*_3_). A crystal suitable for single crystal X-ray diffraction analysis of **20·(PF**_**6**_**)**_**2**_ was grown from diffusion of diethyl ether into an acetonitrile solution of **20·(PF**_**6**_**)**_**2**_.

## Author Contributions

PB conceived of the presented idea. ZL synthesized compounds and recorded NMR spectra. NW recorded and analyzed high resolution mass spectra. PB and ZL collected X-ray diffraction data. All authors discussed the results and contributed to the final manuscript.

### Conflict of Interest Statement

The authors declare that the research was conducted in the absence of any commercial or financial relationships that could be construed as a potential conflict of interest.

## Supplementary Material

Supporting Information Synthetic details for compounds **1**-**3**, Crystallographic data for compounds **5·Br**_**2**_, **9·Br**_**4**_, **10·Br**_**3**_**PF**_**6**_, **11·Br**_**4**_, **15·Br**_**2**_**(PF**_**6**_**)**_**4**_, and **20·(PF**_**6**_**)**_**2**_ and further X-ray crystallographic details and anion binding Job plots for compounds **9·(PF**_**6**_**)**_**4**_ and **10·(PF**_**6**_**)**_**4**_ and 1H and 13C NMR spectra for tetra-imidazolium salts and metal complexes.
