# Supplementary material for: Stepwise Synthesis of Tetra-imidazolium Macrocycles and Their N-Heterocyclic Carbene Metal Complexes
Source: Front Chem. 2019 Apr 24;7:270. doi: 10.3389/fchem.2019.00270 (PMC6491846; doi:10.3389/fchem.2019.00270)
Supplement: Supporting Information — Synthetic details for compounds 1-3, Crystallographic data for compounds 5·Br2, 9·Br4, 10·Br3PF6, 11·Br4, 15·Br2(PF6)4, and 20·(PF6)2 and further X-ray crystallographic details and anion binding Job plots for compounds 9·(PF6)4 and 10·(PF6)4 and 1H and 13C NMR spectra for tetra-imidazolium salts and metal complexes. [file Data_Sheet_1.docx]

Supporting Information for:

Stepwise Synthesis of Tetra-imidazolium Macrocycles and their N-Heterocyclic Carbene Metal Complexes

Zili Li Nuchareenat Wiratpruk and Peter J. Barnard*

Department of Chemistry and Physics, La Trobe Institute For Molecular Science, La Trobe University, Victoria, 3086, Australia, Fax: (+)61 3 9479 1266, E-mail: p.barnard@latrobe.edu.au.

**Synthesis**

**1**. This compound was prepared according to a literature procedure.^21^ A solution of α,α′-dibromo-*o*-xylene (3.00 g, 11.30 mmol) and imidazole (12.38 g, 181.8 mmol) in methanol (100 mL) was stirred at reflux for 27 h followed by evaporation *in vacuo* to obtain a yellow oil. To the resultant residue, a solution of 1.4 M K_2_CO_3_ in aqueous (200 mL) was added and the mixture was stirred overnight. The precipitate was collected and recrystallised in methanol (15 mL) yielding a white solid. Yield: 1.56 g, 57.6%. ^1^H-NMR (500.02 MHz, d_6_-DMSO): δ = 7.71 (s, 2H, *H*_imi_), 7.29 – 7.32 (m, 2H, Ar*H*), 7.09 (t, ^3^*J*_H-H_ = 1.0 Hz 2H, *H*_imi_), 6.96 – 6.98 (m, 2H, Ar*H*), 6.95 (t, ^3^*J*_H-H_ = 1.0 Hz 2H, *H*_imi_), 5.33 (s, 4H, C*H*_2_). ^13^C-NMR (125.74 MHz, d_6_-DMSO): δ = 138.18 (*C*_q_), 135.73 (*C*_imi_), 129.29 (*C*_imi_), 128.72 (*C*_Ar_), 128.45 (*C*_Ar_), 120.23 (*C*_imi_), 46.92 (*C*H_2_). C_14_H_15_N_4_^+^ m/z = 239.1285, calcd = 239.1291.

**2**. This compound was prepared in the same method as **1** according to a literature procedure^21^ by using α,α′-dibromo-*m*-xylene (4.00 g, 15.15 mmol) and imidazole (16.50 g, 242.26 mmol) in methanol (120 mL). Yield: 2.60 g, 72.0%. ^1^H-NMR (500.02 MHz, d_6_-DMSO): δ = 7.74 (s, 2H, *H*_imi_), 7.34 (t, ^3^*J*_H-H_ = 7.5 Hz, 1H, Ar*H*), 7.21 (s, 1H, Ar*H*), 7.15 – 7.16 (m, 4H, Ar*H*, *H*_imi_), 6.91 (s, 2H, *H*_imi_), 5.18 (s, 4H, C*H*_2_). ^13^C-NMR (125.74 MHz, d_6_-DMSO): δ = 138.75 (*C*_q_), 137.87 (*C*_imi_), 129.58 (*C*_Ar_), 129.21 (*C*_imi_), 127.29 (*C*_Ar_), 127.09 (*C*_Ar_), 199.99 (*C*_imi_), 49.75 (*C*H_2_). HR-MS (ESI): calcd for C_14_H_15_N_4_^+^: 238.12, m/z = 239.1240. HRESI-MS^+^ (CH_3_CN): C_14_H_15_N_4_^+^ m/z = 239.1240, calcd = 239.1291.

**3**. This compound was prepared in the same method as **1** according to a literature procedure^21^ by using α,α′-dibromo-*o*-xylene (2.0 g, 8.44 mmol) and 2-methylimidazole (11.09 g, 135.04 mmol) in methanol (100 mL). A solution of 0.5 M K_2_CO_3_ in aqueous (100 mL) was used in this workup. Yield: 1.36 g, 67.3%. ^1^H-NMR (500.02 MHz, d_6_-DMSO): δ = 7.25 (dd, ^3^*J*_H-H_ = 5.7 Hz, ^4^*J*_H-H_ = 3.4 Hz, 2H, Ar*H*), 7.02 (d, ^3^*J*_H-H_ = 1.2 Hz, 2H, *H*_imi_), 6.84 (d, ^3^*J*_H-H_ = 1.2 Hz, 2H, *H*_imi_), 6.54 (dd, ^3^*J*_H-H_ = 5.6 Hz, ^4^*J*_H-H_ = 3.5 Hz, 2H, Ar*H*), 5.24 (s,4H, C*H*_2_), 2.19 (s, 6H, C*H*_3_). ^13^C-NMR (125.74 MHz, d_6_-DMSO): δ = 144.83 (*C*_q_), 135.22 (*C*_q_), 128.27 (*C*_Ar_), 127.26 (*C*_Ar_), 126.44 (*C*_imi_), 120.86 (*C*_imi_), 46.31 (*C*H_2_), 13.04 (*C*H_3_). HRESI-MS^+^ (CH_3_CN): C_14_H_15_N_4_^+^ m/z = 267.1593, calcd = 267.1604.

**X-ray crystallography**

Single crystals suitable for X-ray diffraction studies were grown as follows: **5∙Br_2_** and **9∙Br_4_** diffusion of diethyl ether into an methanol solution of the title compound; **10∙Br_3_PF_6_** diffusion of diethyl ether into an acetonitrile solution of the title compound containing 2 drops of a solution of tetra-*n*-butylammonium bromide in acetonitrile; **11∙Br_4_** slow evaporation of a methanol solution of the title compound; **15∙Br_2_(PF_6_)_4_** diffusion of ethyl acetate into an acetonitrile solution of the title compound; **20∙(PF_6_)_2_** were grown by slow diffusion of diethyl ether into an acetonitrile solution of the titled compound. Crystallographic data for all structures determined are given in Tables S1. For all samples, crystals were removed from the crystallisation vial and immediately coated with paratone oil on a glass slide. A suitable crystal was mounted in Paratone oil on a glass fibre and cooled rapidly to 173 K in a stream of cold N2 using an Oxford low temperature device. Diffraction data were measured using an Rigaku Oxford Diffraction SuperNova X-ray Diffraction System mounted with Mo-Kα λ = 0.71073 Å and Cu-Kα λ = 1.54184. Data were reduced and corrected for absorption using the CrysAlis Pro program. The SHELXL2013-2 program was used to solve the structures with Direct Methods, with refinement by the Full-Matrix Least-Squares refinement techniques on F2. The non-hydrogen atoms were refined anisotropically and hydrogen atoms were placed geometrically and refined using the riding model. Coordinates and anisotropic thermal parameters of all non-hydrogen atoms were refined. All calculations were carried out using the program Olex2. Further XRD details are provided in the Supporting Information. CCDC 1885400-1885405 contains the supplementary crystallographic data for this paper. These data can be obtained free of charge from The Cambridge Crystallographic Data Centre via www.ccdc.cam.ac.uk/data_request/cif.

**Details:**

**5∙Br_2_**: Solved in the monoclinic space group *C*2/*c*. The asymmetric unit contains 0.5 molecules of the title compound.

**9∙Br_4_**: Solved in the monoclinic space group *P*2_1_/*c*. The asymmetric unit contains one molecule of the title compound. A level B Checkcif alert: PLAT410_ALERT_2_B Short Intra H...H Contact H25B ..H32A . 1.88 Ang appears to result from the rigid structure of the tetra-imidazolium molecule causing a close interaction between two adjacent benzylic protons.

**10∙Br_3_PF_6_** Solved in the triclinic space group *P*-1. The asymmetric unit contains 1 molecule of the title compound.

**11∙Br_4_**: Solved in the triclinic space group *P*-1. The asymmetric unit contains 1 molecule of the title compound.

**15∙Br_2_(PF_6_)_4_**: Solved in the triclinic space group *P*-1. The asymmetric unit contains one molecule of the hexanuclear Au(I) complex. The hexanuclear complex carries a 6+ charge and is heavily solvated with both acetonitrile and ethyl acetate. A number of these solvent molecules were located and successfully modelled, however highly disordered solvent molecules were also present, which could not be modelled and the SQUEEZE routine of PLATON was utilized to remove the electron densities resulting from these solvent molecules. The checkcif reports a number of level A alerts associated with the bromide anion (Br2) and the additional residual electron density suggests that this atom is disordered over two closely spaced positions, however attempts to model this disorder were unsuccessful. Similarly, level B alerts are present for disorder associated with one of the PF_6_ anions however, attempts to model this disorder were also unsuccessful. Additional electron density within the cage structure is apparently associated with the 6 closely spaced Au(I) centres. Although disordered solvent and anions are clearly problematic for this structure, no disorder is evident for the hexanuclear Au(I) complex, which is well defined.

**20∙(PF_6_)_2_**: Solved in the triclinic space group *P*-1. The asymmetric unit contains 1 molecule of the title compound.

Table S1. Crystallographic data for compounds **5∙Br_2_**, **9∙Br_4_**, **10∙Br_3_PF_6_**, **11∙Br_4_**, **15∙Br_2_(PF_6_)_4_** and **20∙(PF_6_)_2_**

|  | **5∙Br_2_** | **9∙Br_4_** | **10∙Br_3_PF_6_** | **11∙Br_4_** | **15Br_2_(PF_6_)_4_** | **20∙(PF_6_)_2_** |
| --- | --- | --- | --- | --- | --- | --- |
| Empirical formula | C_18_H_22_Br_4_N_4_ | C_34_H_44_Br_4_N_8_O_2_ | C_36_H_42_Br_3_F_6_N_10_P | C_35_H_44_Br_4_N_8_O | C_108_H_118_Au_6_Br_2_F_24_N_26_O_4_P_4_ | C_42_H_50_Br_2_F_12_N_12_P_2_Pd |
| Formula weight | 614.03 | 916.41 | 999.49 | 912.42 | 3765.78 | 1279.1 |
| Temperature/K | 173.0(10) | 122.96(10) | 130.01(10) | 150.00(10) | 173.00(10) | 100.00(10) |
| Crystal system | monoclinic | monoclinic | triclinic | Triclinic | triclinic | triclinic |
| Space group | *C*2/*c* | *P*2_1_/*c* | *P*-1 | *P*-1 | *P*-1 | *P*-1 |
| a/Å | 14.3347(16) | 12.8710(2) | 10.8599(5) | 11.4962(5) | 14.9369(2) | 13.7023(3) |
| b/Å | 10.6333(7) | 23.9257(4) | 10.9352(5) | 12.8708(5) | 15.2472(2) | 14.0092(3) |
| c/Å | 15.7967(17) | 12.1591(2) | 17.9221(8) | 13.9480(5) | 29.5303(4) | 15.4005(3) |
| α/° | 90 | 90 | 78.193(4) | 104.000(3) | 87.0513(11) | 109.735(2) |
| β/° | 121.734(15) | 91.4195(16) | 84.397(4) | 97.228(4) | 77.1272(12) | 108.553(2) |
| γ/° | 90 | 90 | 85.653(4) | 110.155(4) | 87.0120(11) | 94.107(2) |
| Volume/Å^3^ | 2047.8(4) | 3743.22(11) | 2069.96(16) | 1829.64(14) | 6541.98(16) | 2584.34(10) |
| Z | 4 | 4 | 2 | 2 | 2 | 2 |
| ρ_calc_g/cm^3^ | 1.992 | 1.626 | 1.604 | 1.656 | 1.912 | 1.644 |
| μ/mm^‑1^ | 7.872 | 5.586 | 4.566 | 5.691 | 7.454 | 2.054 |
| F(000) | 1192 | 1840 | 1004 | 916 | 3600 | 1280 |
| Crystal size/mm^3^ | 0.08 × 0.08 × 0.06 | 0.1 × 0.08 × 0.04 | 0.05 × 0.02 × 0.01 | 0.01 × 0.01 × 0.002 | 0.1 × 0.07 × 0.02 | 0.03 × 0.02 × 0.02 |
| Radiation | MoKα (λ = 0.71073) | CuKα (λ = 1.54184) | CuKα (λ = 1.54184) | CuKα (λ = 1.54184) | MoKα (λ = 0.71073) | MoKα (λ = 0.71073) |
| 2Θ range for data collection/° | 6.064 to 52.738 | 7.39 to 130.166 | 8.194 to 148.988 | 6.71 to 130.14 | 5.6 to 60.64 | 6.476 to 49.416 |
| Index ranges | -16 ≤ h ≤ 17, -13 ≤ k ≤ 13, -19 ≤ l ≤ 15 | -15 ≤ h ≤ 13, -28 ≤ k ≤ 18, -14 ≤ l ≤ 14 | -12 ≤ h ≤ 13, -13 ≤ k ≤ 13, -22 ≤ l ≤ 22 | -13 ≤ h ≤ 13, -15 ≤ k ≤ 14, -16 ≤ l ≤ 16 | -19 ≤ h ≤ 21, -20 ≤ k ≤ 21, -41 ≤ l ≤ 41 | -16 ≤ h ≤ 16, -16 ≤ k ≤ 16, -18 ≤ l ≤ 16 |
| Reflections collected | 5867 | 12945 | 19369 | 14785 | 102731 | 29090 |
| Independent reflections | 2094 [R_int_ = 0.0253, R_sigma_ = 0.0303] | 6369 [R_int_ = 0.0218, R_sigma_ = 0.0261] | 8443 [R_int_ = 0.0398, R_sigma_ = 0.0440] | 6244 [R_int_ = 0.0311, R_sigma_ = 0.0389] | 34834 [R_int_ = 0.0320, R_sigma_ = 0.0429] | 8779 [R_int_ = 0.0352, R_sigma_ = 0.0437] |
| Data/restraints/parameters | 2094/0/118 | 6369/0/440 | 8443/0/511 | 6244/0/437 | 34834/1/1576 | 8779/0/646 |
| Goodness-of-fit on F^2^ | 1.062 | 1.034 | 1.033 | 1.035 | 1.036 | 1.068 |
| Final R indexes [I>=2σ (I)] | R_1_ = 0.0373, wR_2_ = 0.0917 | R_1_ = 0.0321, wR_2_ = 0.0845 | R_1_ = 0.0387, wR_2_ = 0.0973 | R_1_ = 0.0301, wR_2_ = 0.0746 | R_1_ = 0.0437, wR_2_ = 0.0970 | R_1_ = 0.0397, wR_2_ = 0.1010 |
| Final R indexes [all data] | R_1_ = 0.0468, wR_2_ = 0.0973 | R_1_ = 0.0353, wR_2_ = 0.0868 | R_1_ = 0.0512, wR_2_ = 0.1059 | R_1_ = 0.0351, wR_2_ = 0.0786 | R_1_ = 0.0639, wR_2_ = 0.1069 | R_1_ = 0.0500, wR_2_ = 0.1053 |
| Largest diff. peak/hole / e Å^-3^ | 1.57/-1.15 | 1.39/-0.49 | 0.95/-0.63 | 1.01/-0.60 | 5.05/-5.20 | 1.42/-1.36 |

**Crystal Structures**

**
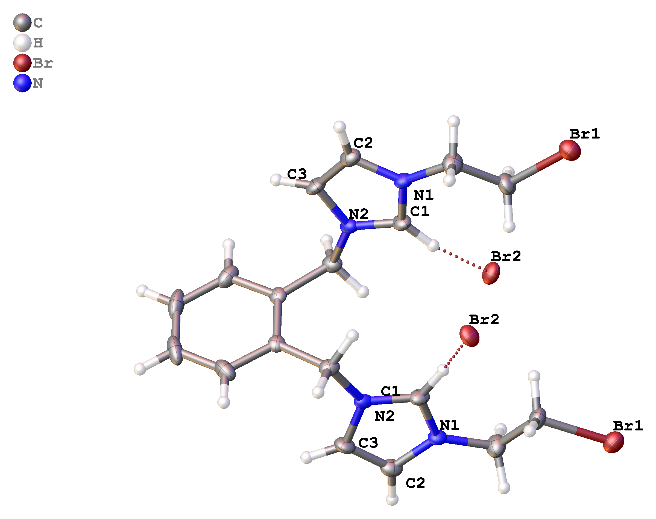
**

Figure S1. Representation of the X-ray crystal structure of **5∙Br_2_**. Atomic displacement ellipsoids are shown at the 50% probability level.

**Anion binding studies**





Figure S2. Experimental titration data (black line, squares) and fitted binding isotherms (red dashed line, circles) for the addition of the Bu_4_N^+^ halide anion (Br^-^ or I^-^) to a solution of **10∙(PF_6_)_4_** in d_6_-DMSO.

**

**

Figure S3. Job plot based on the ^1^H-NMR titration of **9∙(PF_6_)_4_** with Bu_4_N∙Cl, Bu_4_N∙Br and Bu_4_N.I.





Figure S4. Job plot based on the ^1^H-NMR titration of **10∙(PF_6_)_4_** with Bu_4_N∙Cl, Bu_4_N∙Br and Bu_4_N.I.

**^1^H-NMR and ^13^C-NMR spectra of the selected compounds.**

Figure S5. ^1^H-NMR spectrum of pro-ligand **9∙Br_4_**.

Figure S6. ^13^C-NMR spectrum of pro-ligand **9∙Br_4_**.

Figure S7. ^1^H-NMR spectrum of pro-ligand **10∙Br_4_**

**_4_**.

Figure S8. ^13^C-NMR spectrum of pro-ligand **10∙Br_4_**.

Figure S9. ^1^H-NMR spectrum of pro-ligand **11∙Br_4_**.

Figure S10. ^13^C-NMR spectrum of pro-ligand **11∙Br_4_**.

Figure S11. ^1^H-NMR spectrum of pro-ligand **12∙Br_4_**.

Figure S12. ^13^C-NMR spectrum of pro-ligand **12∙Br_4_**.

Figure S13. ^1^H-NMR spectrum of pro-ligand **13∙Br_4_**.

Figure S14. ^13^C-NMR spectrum of pro-ligand **13∙Br_4_**.

Figure S15. ^1^H-NMR spectrum of hexanuclear Ag(I) complex **14∙(PF_6_)_6_**.

Figure S16. ^13^C-NMR spectrum of hexanuclear Ag(I) complex **14∙(PF_6_)_6_**.

Figure S17. ^1^H-NMR spectrum of hexanuclear Au(I) complex **15∙Br_6_**.

Figure S18. ^13^C-NMR spectrum of hexanuclear Au(I) complex **15∙Br_6_**.

Figure S19. ^1^H-NMR spectrum of Pd(II) complex **16∙(PF_6_)_2_**.

Figure S20. ^13^C-NMR spectrum of Pd(II) complex **16∙(PF_6_)_2_**.

Figure S21. ^1^H-NMR spectrum of mononuclear Ag(I) complex **17∙(PF_6_)_3_**

Figure S22. ^13^C-NMR spectrum of mononuclear Ag(I) complex **17∙(PF_6_)_3_**.

Figure S23. ^1^H-NMR spectrum of dinuclear Au(I) complex **18∙(PF_6_)_2_**.

Figure S24. ^13^C-NMR spectrum of dinuclear Au(I) complex **18∙(PF_6_)_2_**.

Figure S25. ^1^H-NMR spectrum of mononuclear Ag(I) complex **19∙(PF_6_)_2_**.

Figure S26. ^13^C-NMR spectrum of dinuclear Ag(I) complex **19∙(PF_6_)_2_**.
